# Characterization of Proline-Rich Antimicrobial Peptides with SbmA Transporter-Dependent and Independent Antimicrobial Activity toward *Klebsiella pneumoniae*

**DOI:** 10.1021/acsinfecdis.6c00142

**Published:** 2026-04-20

**Authors:** Ridhwana M. Appiah, Robert L. Beckman, Christina M. DeBarro, Hanlin Ren, Jennifer S. Brodbelt, Renee M. Fleeman

**Affiliations:** † Division of Immunity and Pathogenesis, Burnett School of Biomedical Sciences, College of Medicine, 124506 University of Central Florida, Orlando, Florida 32827, United States; ‡ Department of Chemistry, 12330 The University of Texas at Austin, Austin, Texas 78712, United States

**Keywords:** *Klebsiella pneumoniae*, PrAMPs, extracellular polysaccharide, biofilms

## Abstract

The rapid emergence of antibiotic resistance in *Klebsiella pneumoniae* is a pressing concern, largely attributed to its rapid development of antibiotic resistance. Our previous work demonstrated that Bac7 (1–35), a proline-rich antimicrobial peptide (PrAMP), displayed potent antimicrobial activity toward *K. pneumoniae* and aggregated with cell-associated polysaccharides produced by this species. Here, we investigated natural PrAMPs from diverse organisms to explore their antimicrobial activity and interactions with *K. pneumoniae* cell-associated polysaccharides. The PrAMPs apidaecin Cd3+, Tur1A, and PR-39 demonstrated activity against all tested strains, with minimum inhibitory concentrations (MICs) ≤ 1 μmol L^–1^, while only Tur1A and PR-39 had biofilm disruption potential. These active PrAMPs shared common structural features, including a proline content above 36% and a net positive charge exceeding +5. Interestingly, both active and inactive PrAMPs aggregated with cell-associated polysaccharides, indicating that the antimicrobial activity and cell-associated polysaccharide aggregation potential are distinct features of PrAMPs. PrAMPs with SbmA transporter-independent activity did not lyse the membrane but caused membrane depolarization, where increased depolarization was observed with colistin-resistant *K. pneumoniae*. Bacterial mutants lacking LPS modifications conferring colistin resistance displayed rapid uptake of BODIPY-labeled PR-39 and Bac7 (1–35), whereas mutants lacking the SbmA transporter displayed less uptake of PR-39 than Bac7 (1–35). Overall, these findings highlight peptide charge as a critical determinant of membrane interaction and membrane-mediated uptake while also revealing mechanistic insight into how PrAMPs engage with oligosaccharides.

## Graphical abstract

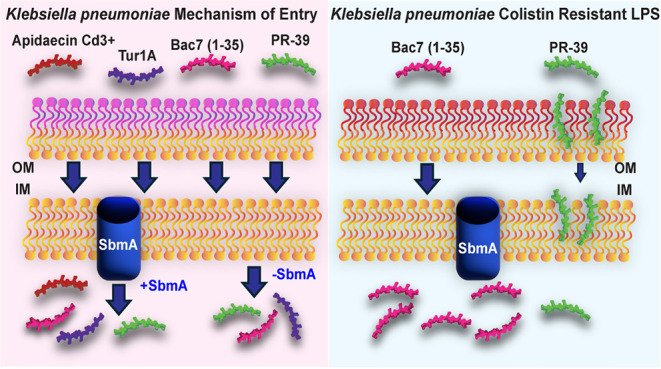

## Introduction

*Klebsiella pneumoniae* is a multidrug-resistant (MDR) pathogen that is on the WHO top priority pathogen list for species of urgent need of novel therapies.^1^ It is a leading cause of hospital-acquired infections, including pneumonia, bloodstream infections, and urinary tract infections, with mortality rates reaching up to 50% even with antibiotic treatments.^2–4^ Unfortunately, this pathogen has acquired multiple genetic elements that allow it to cause community-acquired infections.^5,6^ Traditionally, virulence leading to community-acquired infections was linked to hypervirulent *K. pneumoniae* (hvKp) and MDR to classical *K. pneumoniae* (cKp).^7,8^ However, recent clinical evidence shows a concerning overlap of these traits, complicating treatment and increasing challenges in managing *K. pneumoniae* infections.^9–12^ Adding to this complexity, *K. pneumoniae* displays remarkable genetic diversity and has been reclassified into three phylogroups: KpI (*K. pneumoniae*), KpII (*K. quasipneumoniae*), and KpIII (*K. variicola*).^13,14^ These species vary in their ecological niches, virulence potential, and antibiotic resistance gene profiles, making diagnosis, treatment selection, and infection control increasingly difficult.^15–17^

Antimicrobial peptides (AMPs) are an attractive alternative to small molecule antibiotics for MDR Gram-negative pathogens.^18–20^ Of particular interest are non-lytic PrAMPs that have an intracellular mechanism of action, and their high proline content provides protection from proteolytic degradation of the arginine residues.^21–23^ PrAMPs exhibit antimicrobial activity by passing through the lipopolysaccharide (LPS) outer membrane and utilizing the inner membrane transporter SbmA, contributing to their non-lytic mechanism of action.^24,25^ In our previous study, we found that the PrAMP Bac7 (1–35) exhibited potent antimicrobial activity against *K. pneumoniae*, aggregated strongly with cell-associated polysaccharides, and disrupted preformed biofilms.^26,27^ Moreover, Bac7 (1–35) treatment markedly reduced bacterial burden in a murine skin abscess model.^26,28^ These compelling findings prompted us to explore a broader repertoire of PrAMPs derived from diverse natural host organisms.

Here, we investigated PrAMPs from mammalian and insect origins to assess the diversity of their antimicrobial activity and potential mechanisms of *K. pneumoniae* cell entry. Among the peptides tested, apidaecin Cd3^+^, PR-39, and Tur1A exhibited the strongest antimicrobial activity, although only PR-39 and Tur1A could disrupt preformed biofilms. Notably, the mammalian PrAMPs PR-39 and Tur1A, with increased sequence similarity to Bac7 (1–35) compared to the other PrAMPs tested, induced membrane depolarization without causing cell lysis, suggesting membrane-mediated uptake. C-terminal BODIPY-tagged PR-39, compared to Bac7 (1–35), displayed decreased uptake into the cytosol of colistin-resistant MKP103, which was further impacted when testing a mutant lacking the SbmA transporter, while both Bac7 (1–35)-BODIPY and PR-39-BODIPY displayed increased uptake when testing a mutant lacking colistin-resistant LPS modifications. Collectively, these findings highlight that nonlytic membrane penetration mechanisms used by mammalian PrAMPs increase their antimicrobial activity toward multidrug-resistant *K. pneumoniae* but increase their susceptibility to colistin-resistant LPS modifications.

## Results

### PrAMPs with Sequence Similarity Have a Broad-Spectrum and SbmA Transporter-Independent Antimicrobial Activity

Our previous work revealed that Bac7 (1–35) exhibited potent antimicrobial activity against *K. pneumoniae*. To expand on our findings and understand whether other PrAMPs have similar antimicrobial potency, we evaluated naturally occurring PrAMPs from diverse organisms for their antimicrobial activity against various bacterial strains. We tested PrAMPs that varied in key peptide characteristics, including sequence, length, charge, proline and arginine content, and the presence of proline-arginine-proline (PRP) motifs (Table 1).^29^ Furthermore, the PrAMPs were produced by a variety of organisms ranging from mammals (cow, pig, and dolphin) to insect organisms^30,31^ (Table 2). We tested the PrAMPs against a panel of *K. pneumoniae* isolates including a type strain (ATCC 13883), two hvKp strains (NTUH-K2044 and ATCC 43816), two MDR cKp strains (ATCC 700603 and MKP103), and two non Kp control isolates (*Escherichia coli* and *Acinetobacter baumannii*).

**Table 1:** Selected PrAMPs and Their Key Physiological Characteristics

PrAMPs	Sequence	Length	Charge	%Pro	%Arg	PRP motif
Bac7 (1–35)	RRIRPRPPRLPRPRPRPLPFPRPGPRPIPRPLPFP	35	11	46	31	6
PR-39	RRRPRPPYLPRPRPPPFFPPRLPPRIPPGFPPRFPPRFP	39	10	49	26	3
Tur1A	RRIRFRPPYLPRPGRRPRFPPPFPIPRIPRIP	32	10	38	31	1
arasin 1	SRWPSPGRPRPFPGRPKPIFRPRPC	25	7	36	24	2
apidaecin Cd3+	GKPSKPRPAPIKPRPPHPRL	20	6	40	15	2
oncocin	VDKPPYLPRPRPPRRIYNR	19	5	32	26	2
drosocin	GKPRPYSPRPTSHPRPIRV	19	5	32	21	3
abaecin	YVPLPNVPQPGRRPFPTFPGQGPFNPKIKWPQ	32	4	32	6	0
riptocin	VDKGGYLPRPTPPRPVYRS	19	3	26	16	2
pyrrhocoricin	VDKGSYLPRPTPPRPIYNRN	20	3	25	15	2
metalnikowin	VDKPDYRPRPRPPNM	15	2	33	20	2

**Table 2:** Host Origins for PrAMPs Used in This Study

PrAMPs	Host Organism	Scientific Class
apidaecin Cd3+	Parasitic wasp	Insect
abaecin	Honeybee	Insect
drocosin	Fruit fly	Insect
pyrrhocoricin	Fire beetle	Insect
metalnikowin	Green shield bug	Insect
oncocin	Milkweed bug	Insect
riptocin	Bean bug	Insect
arasin 1	Spider crab	Malacostraca
PR-39	Porcine	Mammal
Tur1A	Dolphin	Mammal
Bac7 (1–35)	Cow	Mammal

Minimum inhibitory concentration (MIC) assays were performed using the antimicrobial peptide-specific broth dilution method described by Wiegand et al., diluting the peptides in a buffer (0.01% acetic acid with 0.2% BSA) to prevent peptide binding to the plastic 96-well plate and increase the efficiency of determining antimicrobial peptide MICs.^32^ Our analysis revealed *E. coli* W3110 was the most sensitive to the PrAMPs compared to *K. pneumoniae* strains, while the nonenteric bacterium *A. baumannii* was the least sensitive to the PrAMPs (Figure 1A). Furthermore, we found that many PrAMPs had no antimicrobial activity toward *K. pneumoniae* and active peptides were distinguished by a significantly higher proline content and a high positive charge compared to moderately active and inactive peptides. Specifically, apidaecin Cd3^+^, PR-39, and Tur1A (charge > +6) exhibited the strongest antimicrobial activity toward all *K. pneumoniae* strains tested (MICs < 1 μmol L^–1^). Interestingly, apidaecin Cd3+, which was isolated from the parasitic wasp *Coccygomimus disparis* has increased charge (+6 vs +3 charge) and PRP motifs (2 vs 1) compared to the well-studied apidaecin 1b isolated from a honey bee (*Apis mellifera*),^29,33,34^ potentially explaining its excellent activity toward *K. pneumoniae*. Nonlytic AMPs, such as PrAMPs, are reported to utilize the proton motive force-dependent membrane transporter SbmA/BacA in genera such as *Mycobacterium*, *Brucella*, and *Sinorhizobium*.^35–37^ To explore PrAMP uptake mechanisms, we tested an SbmA transporter-deficient transposon mutant of the MDR MKP103 strain, MKP103 Δ*sbmA* (KPNIH1–05310–803:T_30_),^38^ and MICs revealed that Bac7 (1–35), PR-39, and Tur1A retained partial activity, showing a respective 8-, 16-, and 8-fold increase in MIC. On the other hand, while apidaecin Cd3+ displayed similar antimicrobial activity toward the parental isolate, it completely lost activity toward MKP103 Δ*sbmA*. To further assess the peptide antimicrobial activity of apidaecin Cd3^+^, PR-39, and Tur1A under different physiological conditions, we performed MICs at room temperature in Mueller Hinton 1 broth (MHB1) and phosphate buffered saline (PBS), to assess the impact of the lower temperatures that cause increased membrane rigidity and if they have activity toward stationary phase cells, respectively.^39,40^ After 48 h of incubation, apidaecin Cd3+ and Tur1A displayed little change in MICs at room temperature (Figure S1A). In contrast, PR-39 exhibited reduced antimicrobial activity at room temperature, with a 2-fold increase in MIC for ATCC 700603 and a 32-fold increase for colistin-resistant MKP103, suggesting that membrane dynamics may impact the antimicrobial activity of PR-39. When assessing minimum bactericidal concentration (MBC) in PBS, PR-39 had an MBC of <0.42 μmol L^–1^, while Tur1A and apidaecin Cd3+ displayed MBC values of 16 μmol L^–1^ and 60.7 μmol L^–1^, respectively (Figure S1B and S1C). When we aligned the PrAMP secondary structures using MEGA, we found that the most active PrAMPs (Bac7 (1–35), PR-39, and Tur1A) have the highest sequence similarity, as can be expected with their shared mammalian origins, but also considering that a pig (PR-39) and dolphin (Tur1A) share a common ancestral lineage predating the evolutionary divergence with the cow that produces Bac7 (1–35) (Figure 1B).^31^

**Figure 1:**
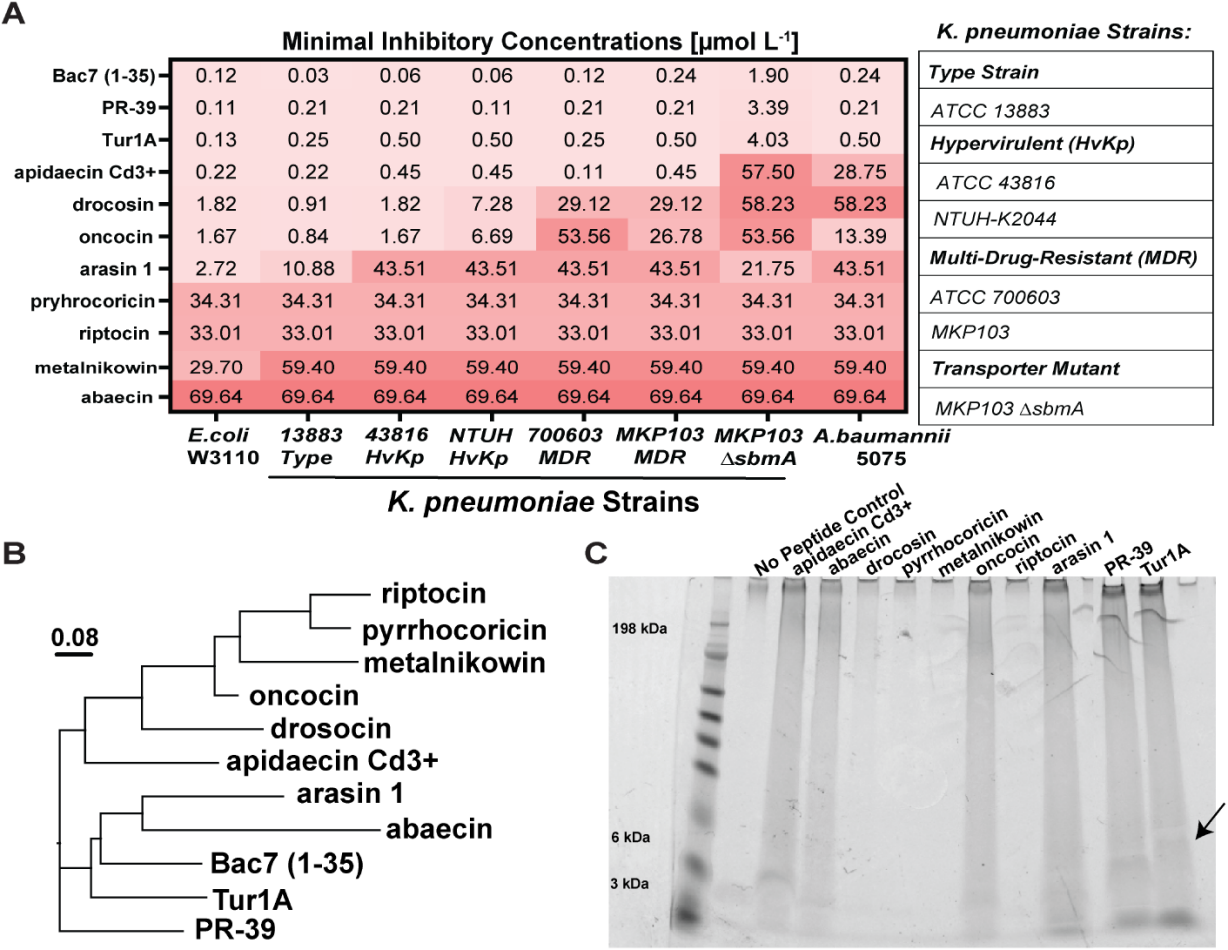
PrAMP with sequence similarity has broad-spectrum and SbmA transporter-independent antimicrobial activity. The figures show the antimicrobial activity, sequence similarity, and aggregation with cell-associated polysaccharides of natural PrAMPs from diverse organisms. Figure 1A shows the MICs (μmol L^‑1^) of PrAMPs toward *E. coli*, *A. baumannii* 5075, and a panel of *K. pneumoniae* isolates: Kp type strain (13883), 2 hvKp strains (NTUH-K2044 and 43816), and 2 MDR cKp strains (700603 and MKP301). The MDR Kp MKP103 was the parental strain for the transporter mutant (ΔsbmA). Figure 1B shows the sequence similarity between the PrAMPs tested using MEGA software and MAFFT parameters with neighbor-joining distance displayed. Figure 1C shows PrAMPs (100 μmol L^‑1^) aggregation with cell-associated polysaccharide (100 μg mL^‑1^) on a 4–8% bis-tris SDS-PAGE gel stained with Alcian blue. The top bands (>198 kDa) correspond to the clonal CPS (CPS lower molecular weight appears as a smear), and lower bands (3 kDa and 6 kDa) correspond to the core and lipid A LPS components, respectively. No peptide treatment with cell-associated polysaccharides only was used as a control (No Peptide Control) and run alongside the aggregates to account for polysaccharide binding to the microcentrifuge tubes. Arrows on the SDS-PAGE gel show the shift in core LPS compared to the no peptide control. All MICs and cell-associated polysaccharide aggregation gels shown were a result of biological triplicate assessments, with a representative SDS-PAGE gel shown.

We have previously shown that host defense peptide aggregation with purified cell-associated polysaccharides containing both capsular polysaccharides (CPS) and lipopolysaccharides (LPS) correlates with their antimicrobial activity toward *K. pneumoniae* and Bac7 (1–35) displays significantly more aggregation than other host defense peptides.^26,27,41^ Therefore, PrAMPs were added to the purified cell-associated polysaccharides from MKP103, the strain that had the highest MIC values. The samples were centrifuged to precipitate the aggregates next to a cell-associated polysaccharide control sample without PrAMPs (Figure S2A). Predominantly, we found PrAMPs that formed detectable aggregates with polysaccharides had a net positive charge above +4 (Table 1). Furthermore, Tur1A and PR-39 displayed aggregation potential similar to what was observed with Bac7 (1–35) in our previous work.^26^ The polysaccharides (CPS and LPS) that aggregated with the PrAMPs were visualized on an SDS-PAGE gel next to the control sample without PrAMPs, validating that Tur1A and PR-39 had the greatest aggregation potential (Figure 1C). However, not all PrAMP aggregation aligned with antimicrobial activity, as previously described with α-helical, membrane-disrupting peptides.^41^ Specifically, the inactive PrAMP abaecin aggregated with polysaccharides, while the active PrAMP drocosin did not. We found that the smearing on the SDS-PAGE gel characteristic of lower molecular weight CPS, was only visible with the highest abundance of polysaccharide on the gel, in line with the finding that these are a minor component of the capsule.^42,43^ Furthermore, we found that the LPS bands (6 kDa core LPS; 3 kDa LPS lipid A) shifted in migration with PR-39 and Tur1A, indicating potential increased binding to LPS. We quantified the CPS (>198 kDa only, without smear) and LPS (6 kDa and 3 kDa) bands using ImageJ and found that PR-39 and Tur1A had the greatest band density of PrAMPs (Figure S2B–S2D). Together, these findings illustrate the broad spectrum of PrAMPs’ antimicrobial activity toward *K. pneumoniae*, and although polysaccharide aggregation is possible with inactive PrAMPs, the analogs that are active in the absence of the SbmA transporter strongly associate with the LPS.

### Transporter-Independent PrAMPs Display Biofilm Disruption Potential

Our previous studies demonstrated that Bac7 (1–35) induces biofilm disruption,^26^ providing insight that PrAMPs can target biofilm-associated infections. Based on the ability of Bac7 (1–35) to disrupt biofilms, we hypothesized that other PrAMPs could similarly affect biofilm integrity. We assessed the viability of cells within the biofilms of PrAMP-treated cultures and visualized the changes to the cell population and polysaccharide matrix by using confocal microscopy. Biofilms were formed with *K. pneumoniae* NTUH-K2044, which was the strongest biofilm former in the study, and we transformed it with pMF230_GFP to perform downstream confocal imaging of the biofilms.

We treated preformed biofilms for 24 h with 30 μmol L^–1^ of PrAMPs that have (Bac7 (1–35), PR-39, and Tur1A) and do not have (apidaecin Cd3+, arasin 1, and oncocin) SbmA-independent killing, enumerated biofilm cell viability, and performed confocal z-stack imaging to evaluate biofilm 3D spatial matrix changes and 2D cell density post-treatment. When testing cell viability, we found that peptides with increased positive charge, which bypass the SbmA transporter, can also more effectively disrupt attached biofilms, although to different extents (Figure 2A). Specifically, PR-39 had the most significant reduction in cell viability from the no treatment samples, and Tur1A did not have a significant reduction. Cells dispersed from the biofilm into the culture supernatant showed a significant decrease in viability following treatment with all three SbmA-independent killing PrAMPs (Figure 2B). To observe changes in biofilm matrix height with these treatments, we performed confocal z-stack imaging to visualize the cellular population by GFP expression and polysaccharides with Texas Red-conjugated concanavalin A, a lectin that binds specifically to alpha-mannopyranosyl and alpha-glucopyranosyl residues.^44,45^ Untreated biofilms had an average height of ∼35 μm, with PR-39 and Tur1A-treated biofilms displaying a reduction in biofilm height (Figure 2C) and GFP-expressing biofilm-associated cells (Figure 2D). Treatment using the less active, transporter-dependent apidaecin Cd3+, oncocin, abaecin, and arasin-1 did not cause a reduction in polysaccharide matrix height or biofilm-associated cells in comparison to the untreated controls (Figure S3). These findings indicate that PrAMPs with increased antimicrobial activity and transporter-independent killing can also decrease biofilm-associated cell populations.

**Figure 2:**
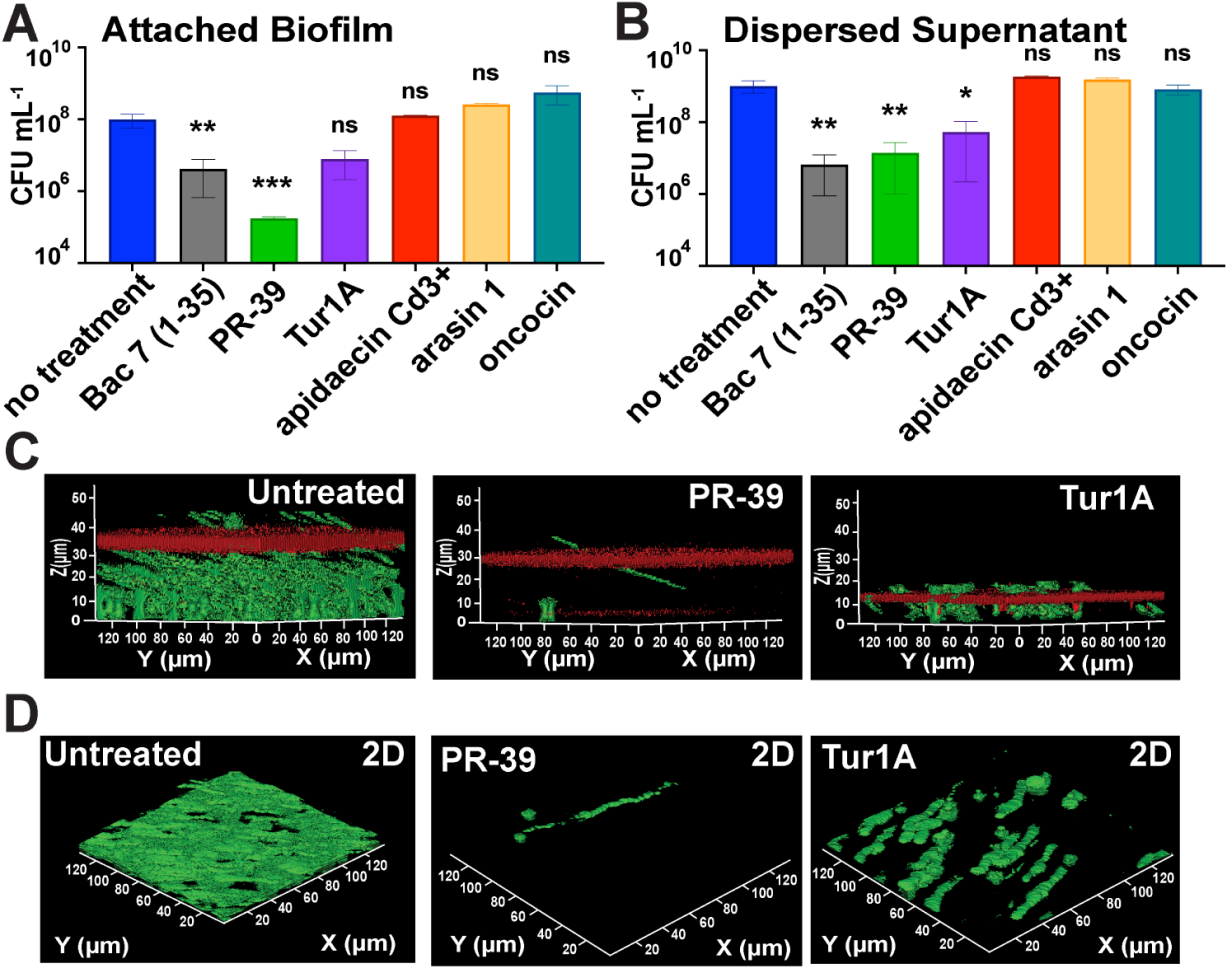
Transporter-independent PrAMPs display a biofilm disruption potential. The figures show cell viability with and without treatment of preformed biofilms with 30 μmol L^‑1^ PrAMPs and confocal z-stack imaging with NTUH-K2044 constitutively expressing GFP to show the cell population as green, and the polysaccharide matrix is stained red with concanavalin A conjugated to Texas Red. Figure 2A is the cell viability of the attached biofilm cells, and Figure 2B shows the cell viability of the dispersed supernatant. Figure 2C shows the 3D rendering of confocal z-stack imaging of untreated biofilms next to biofilms treated with PR-39 and Tur1A to reveal the changes in height of the biofilms. Figure 2D shows 2D images of untreated biofilms next to biofilms treated with PR-39, Tur1A, apidaecin Cd3+ (apidaecin), oncocin, and arasin 1 (arasin), with only the green fluorescence shown to reveal the changes in biofilm-associated cell density following treatment. Triplicate biofilms were imaged, with representative images shown. Triplicate biofilms were enumerated for Figures 2A and 2B, with error shown as as ±SEM and significance determined using one-way ANOVA with Dunnett’s correction for multiple comparisons (* p-value < 0.05, ** p-value < 0.01, and *** p-value < 0.001).

### Membrane Depolarization by Tur1A and PR-39 Suggests Nonlytic Membrane Translocation

PrAMPs have been reported to be nonlytic but have multiple mechanisms of action depending on the bacterial genera.^21,23,46^ We previously published that Bac7 (1–35) causes significant membrane depolarization of *K. pneumoniae* membranes when penetrating the cell without causing cell leakage.^47^ Considering PR-39 and Tur1A, which displayed antimicrobial activity toward the SbmA transporter-deficient transposon mutant like Bac7 (1–35) (Figure 1), we wanted to assess the contribution of membrane interactions to their antimicrobial activity. For a thorough analysis, we first validated that the PrAMPs were nonlytic using leakage of large and small molecules (o-nitrophenyl-β-D-galactoside (ONPG) and ATP, respectively) and then determined their impact on membrane polarity using DiSC_3_(5), a cationic hydrophobic dye.^48,49^

To validate the nonlytic nature of Tur1A and PR-39, we used MDR ATCC 700603 to perform an inner membrane permeability assay using ONPG, a substrate for cytoplasmic β-galactosidase naturally produced by *K. pneumoniae* ^50,51^ and an ATP leakage assay to measure small metabolite leakage.^52,53^ As expected, when measuring the leakage of large molecules, melittin, a known membrane-lytic peptide, exhibited a significant increase in o-nitrophenol absorbance compared to the untreated control (Figure 3A). In contrast, Tur1A and PR-39 (1 μmol L^–1^) showed no difference in o-nitrophenol absorbance compared to the control. We then assessed leakage of small metabolites by measuring ATP in filter-sterilized supernatants of treated cultures (1 μmol L^–1^) using the BacTiter-Glo kit. We found minimal changes in supernatant levels of ATP with PrAMP treatment (Figure 3B), validating that they do not cause leakage of small metabolites. To understand the changes to the membrane potential, we used a kinetic assay to follow the quenching of DiSC_3_(5) dye before and after treatment with PR-39 and Tur1A, which are active in the absence of the transporter, next to transporter-dependent apidaecin Cd3+. When testing MDR ATCC 700603 (cKp) with 1 μmol L^–1^ of the PrAMPs next to the polymyxin B control membrane-lytic peptide, we found that PR-39 and Tur1A, but not apidaecin Cd3+, displayed rapid membrane depolarization following the addition of the peptides (Figure 3C). However, PR-39 induced sustained depolarization throughout the assay, whereas Tur1A-mediated depolarization diminished over time. We then tested the more PrAMP-sensitive NTUH-K2044 (hvKp) and found minimal membrane depolarization (Figure 3D). Comparing the fold change in fluorescence intensity between the fully quenched dye (30 min) and the peak fluorescence following PrAMP addition (40 min), we found that only PR-39 and Tur1A treatment resulted in different depolarization between MDR ATCC 700603 (cKp) compared to non-MDR NTUH-K2044 (hvKp) (Figure 3E). We assessed cell recovery following these assays and found that PR-39 had an increased rate of killing at higher concentrations, regardless of pathotype, when compared to Tur1A and apidaecin Cd3+ (Figure S4).

**Figure 3:**
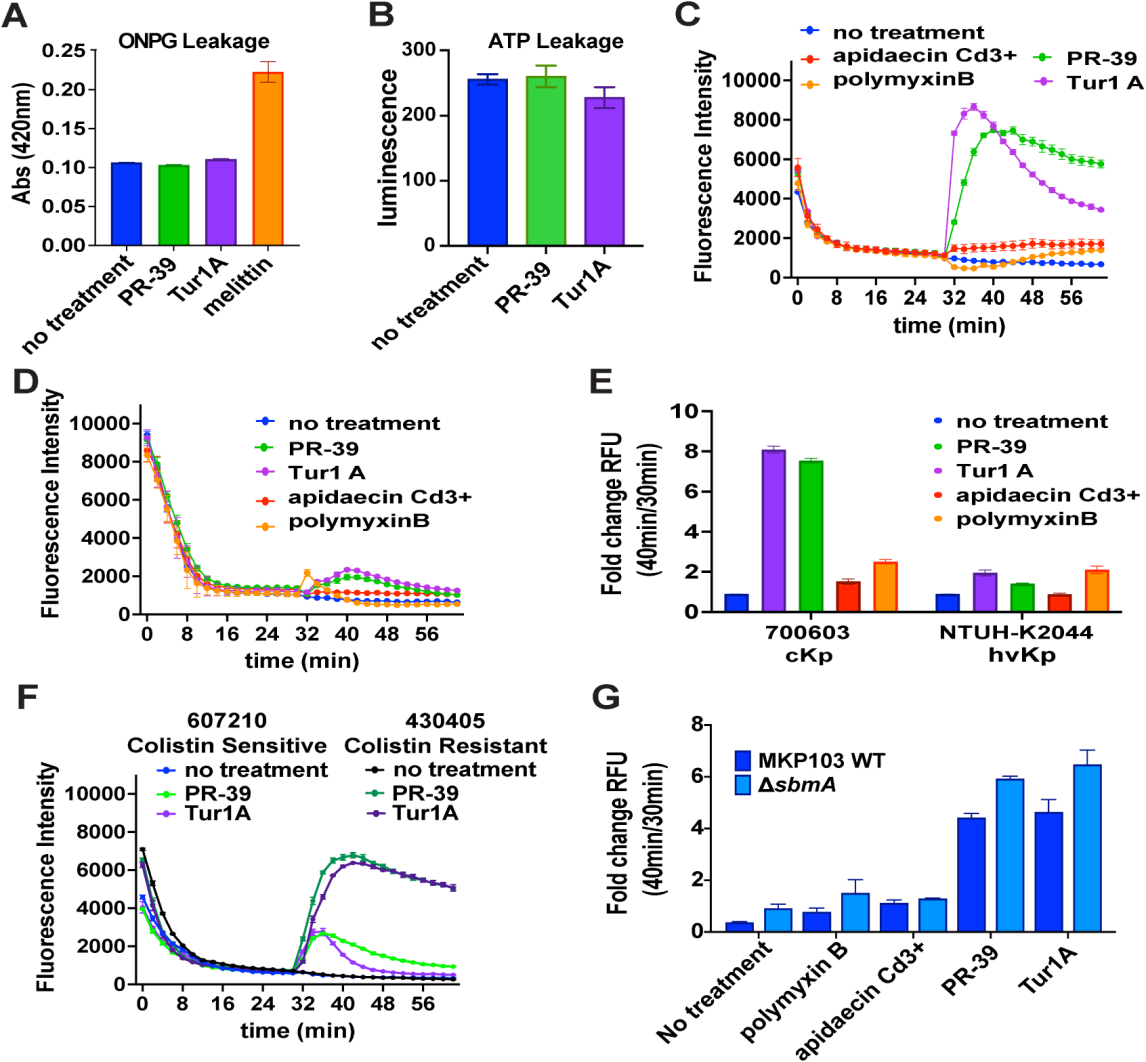
Membrane depolarization by Tur1A and PR-39 suggests nonlytic membrane translocation. The figures show membrane leakage and depolarization assays with no treatment or 1 μmol L^‑1^ of PR-39, Tur1A, apidaecin, Cd3+, and polymyxin B. Figure 3A shows leakage of cytoplasmic β-galactosidase from MDR 700603 with PrAMP treatment using ONPG as the substrate and following the conversion to the end product o-nitrophenol (ONP), detected over 45 min using absorbance at 420 nm. Figure 3B shows leakage of ATP from the cytosol using a BacTiter-Glo kit to measure luminescence of filter-sterilized supernatants of MDR 700603 following 1 h treatment with or without PrAMPs. Figure 3C and 3D show the kinetic assays for MDR 700603 and hvKp NTUH-K2044, respectively, following DiSC3 fluorescence quenching for the first 30 min, where the peptide is added and kinetic measurements continue for an additional 30 min. Figure 3E shows the fold change between peak fluorescence read after PrAMP addition (40 min) and the fully quenched dye fluorescence before PrAMP addition (30 min) for both MDR 700603 and hvKp NTUH-K2044. Figure 3F shows the DiSC3 kinetic assay for colistin-sensitive (MRSN 607210) and colistin-resistant (MRSN 430405) clinical isolates. Figure 3G shows the fold change between peak fluorescence read after PrAMP addition (40 min) and the fully quenched dye fluorescence before PrAMP addition (30 min) for colistin-resistant MKP103 and MKP103 ΔsbmA. All data are reported from biological triplicate assessments, and error is represented as ±SEM.

To understand these findings in the context of clinical isolates with different membrane-mediated colistin resistance, we performed DiSC_3_(5) kinetic analysis on clinical isolates MRSN 430405 and MRSN 607210 (colistin MICs of 14 μmol L^–1^ and 1 μmol L^–1^, respectively)­(Figure S5A and S5B) and found increased, sustained depolarization with MRSN 430405 compared to the colistin-sensitive MRSN 607210 (Figure 3F). Interestingly, Tur1A and PR-39 have increased MICs toward MRSN 430405 (2.0 and 1.7 μmol L^–1^, respectively) compared to MRSN 607210 (1.0 and 0.8 μmol L^–1^, respectively), revealing a similar inverse correlation with colistin and membrane depolarization that we found with Bac7 (1–35).^47^ When testing the colistin-resistant MKP103 and MKP103 Δ*sbmA*, we found PR-39 and Tur1A displayed increased membrane depolarization with minimal DiSC_3_(5) requenching compared to apidaecin Cd3+ (Figure S5C and S5D). We saw an increased fold change in peak fluorescence (40/30 min) with PR-39 and Tur1A treatment of MKP103 Δ*sbmA* compared to the parental isolate (Figure 3G). The decreased antimicrobial activity of PR-39 and Tur1A toward MKP103 Δ*sbmA,* combined with the relatively higher depolarization, confirms membrane interactions by PrAMPs do not directly contribute to killing and reveals that membrane penetration may be a less efficient cell uptake mechanism. Collectively, these findings validate PrAMPs’ nonlytic mechanism of killing and reveal that increased membrane depolarization correlates with decreased antimicrobial activity across *K. pneumoniae*.

### Colistin Resistance Impacts PrAMP Rate of Killing and Membrane Translocation

We have shown that there are two distinct mechanisms of *K. pneumoniae* cell entry by Bac7 (1–35),^47^ PR-39, and Tur1A. Membrane translocation allows for continued activity with loss of the SbmA transporter, potentially decreasing the ability of *K. pneumoniae* to develop resistance to these PrAMPs. However, the increased depolarization observed with colistin-resistant isolates suggests that membrane-mediated resistance can also impact PrAMP antimicrobial activity. To test the impact that colistin-resistant LPS modifications have on antimicrobial activity and cell entry compared to the loss of the SbmA transporter, we used colistin-resistant ST258 MKP103 (colistin MIC 64 μmol L^–1^),^54^ next to mutants lacking transporter activity (MKP103 Δ*sbmA*) and LPS colistin-resistant modifications (MKP103 Δ*phoP* (KPNIH1_10030–701:T30)).

To assess changes in killing potential, we performed a time-kill assessment using the colistin-resistant MKP103 parental isolate, MKP103 Δ*sbmA*, and MKP103 Δ*phoP* using spot plating to determine the minimal bactericidal concentrations (MBC) after 30 min, 1, 2, and 24 h of growth in MHB1 (Figures S6–S7). Overall, we found that the PrAMPs displayed bacteriostatic activity at early time points with the parental isolate and MKP103 Δ*sbmA*, with bactericidal activity only after 24 h. However, with MKP103 Δ*phoP* we saw bactericidal activity at early time points, highlighting the importance of the PhoPQ system in decreasing the rate of PrAMPs’ bactericidal activity.^55^ Specifically, MKP103 Δ*phoP* displayed increased sensitivity to all PrAMPs after 2 h compared to the parental strain (Fold Change in MBC > 2), while there were minimal changes with MKP103 Δ*sbmA* (Figure 4A). We found that after 24 h of incubation with PrAMPs, there was less of a difference in MBCs between the MKP103 parental and *phoP* mutant (Fold Change in MKP103 Δ*phoP* MBC ≤2) (Figure 4B). Conversely, 24 h of incubation with PrAMPs resulted in much higher MBCs with MKP103 Δ*sbmA* compared to the parental strain (Fold Change in Δ*sbmA* MBC ≤ 0.5), indicating that SbmA is still the preferred route of entry even with transporter-independent killing PrAMPs. Interestingly, PR-39 displayed the least change in MBC values between MKP103 parental and MKP103 Δ*sbmA,* while apidaecin Cd3+ displayed the lowest MBCs toward the parental isolate and had no MBC activity toward MKP103 Δ*sbmA*. These results show that the SbmA transporter is more important for the overall killing potential of PrAMPs, while the *K. pneumoniae* PhoP membrane modifications impact the rate of bactericidal activity.

**Figure 4:**
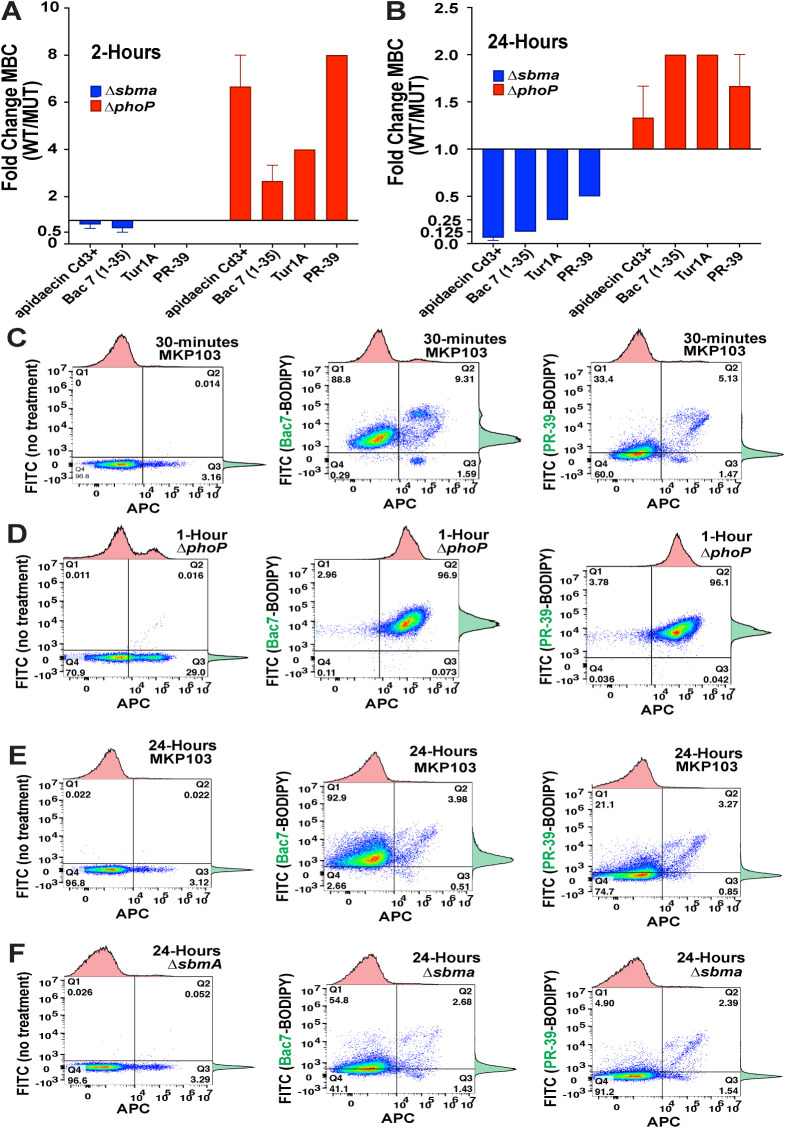
Colistin resistance impacts the PrAMP rate of killing and membrane translocation. The figures show the fold change in bactericidal activity and differential peptide uptake between colistin-resistant MKP103, MKP103 ΔsbmA, and MKP103 ΔphoP. Figure 4A shows the fold change in MBC values between MKP103 and mutants (ΔsbmA and ΔphoP) after 2 h of growth in MHB1. Figure 4B shows the fold change in MBC values between MKP103 and mutants (ΔsbmA and ΔphoP) after 24 h of growth in MHB1. Figure 4C shows pseudo-color dot plots and associated histogram plots of no treatment next to Bac7-BODIPY and PR-39-BODIPY cell uptake by MKP103 using flow cytometry with FITC (green histogram) and APC lasers (red histogram) to detect labeled PrAMPs and bacterial cells, respectively. Figure 4D shows no treatment next to Bac7-BODIPY and PR-39-BODIPY cell uptake by MKP103 ΔphoP. Figures 4E and 4F show no treatment next to Bac7-BODIPY and PR-39-BODIPY cell uptake by MKP103 parental and MKP103 ΔsbmA, respectively, after 24 h. Quadrants are used to show cells that take up low or high amounts of BacLight Red cell stain (Q4 and Q3, respectively) and cells that take up the BODIPY-tagged PrAMPs (Q1 and Q2). All data are reported from biological triplicate assessments, with error represented as ±SEM for Figure 4A and 4B, and representative FlowJo pseudo-color dot plots shown for Figure 4C–4F. All quadrant quantification data from triplicate experiments is shown in Figure S8.

To understand how Bac7 (1–35) and PR-39 differential MBCs correlate with uptake, we treated the MKP103 parental isolate, MKP103 Δ*sbmA*, and MKP103 Δ*phoP* with C-terminal BODIPY-tagged peptides to allow us to measure peptide uptake in the population by flow cytometry. We incubated 4 μmol L^–1^ of Bac7 (1–35)-BODIPY and PR-39-BODIPY with MKP103 parental and mutant strains, followed by peptide removal and staining of the bacterial cells with BacLight^TM^ red cell stain. We used a gating strategy to quantify the number of cells with high peptide uptake and low permeability (Q1), high peptide uptake and high permeability (Q2), no peptide uptake and high membrane permeability (Q3), and no peptide uptake with low membrane permeability (Q4) from triplicate experiments (Figure S8) with representative pseudo-color dot plots shown (Figure[Fig fig4]C–F). With 30 min-incubation, we found that Bac7 (1–35)-BODIPY was taken up more efficiently into the parental strain (88.8% Quadrant 1), while PR-39-BODIPY displayed comparatively less uptake (33.4% Quadrant 1 and 60% Quadrant 4) (Figure 4C). Intriguingly, we saw a subpopulation of cells that had more PrAMP uptake and increased membrane dye binding (Quadrant 2), which indicates increased membrane permeability or cell death within this subpopulation. Interestingly, the entire population of MKP103 Δ*phoP* cells displayed equally increased peptide uptake and membrane permeability (Figure 4D), which correlates with an overall increased rate of killing in MKP103 Δ*phoP*. When assessing 24 h of incubation, we saw similar uptake with the parental isolate as observed at 30 min, where Bac7-BODIPY was taken up to a greater extent (Figure 3E). Finally, in line with the loss of antimicrobial activity across all PrAMPs with deletion of the SbmA transporter, we found MKP103 Δ*sbmA* to have less overall uptake of both BODIPY-tagged peptides (Figure 3F) at all time points tested (Figure S8). Together these findings highlight that PrAMP membrane translocation to the cytosol induces depolarization, and increased membrane interactions with PR-39 decrease the rate of peptide uptake in colistin-resistant *K. pneumoniae* compared to Bac7 (1–35).

### Tur1A Displays Increased Interactions with Hyaluronic Acid

Our previous work with Bac7 (1–35) revealed distinct residues within the peptide’s secondary amino acid sequence that are involved with complexing with polysaccharides.^26^ Native mass spectrometry (ESI-MS) provides a platform that can preserve and detect noncovalent peptide•ligand complexes in the gas phase. Subsequent activation of the complexes using ultraviolet photodissociation (UVPD) cleaves the peptide backbone and creates apo (ligand-free) and holo (ligand-retaining) fragment ions, which allow localization of the peptide•ligand interactions. With our intriguing results of the strong membrane interactions and the variability in LPS binding between PR-39 and Tur1A, we wanted to use ESI-MS and perform UVPD on these peptides to determine whether they bind similarly to tetrasaccharides or if the minor variations in their secondary structure change their interactions with tetrasaccharides. Due to the complex nature of extracted LPS, we examined two polysaccharide surrogates: hyaluronic acid (negatively charged) and stachyose (neutral), the latter of which was used in our previous work.^26^

The formation of 1:1 complexes between peptides (PR-39, Tur1A, and Bac7 (1–35)) and tetrasaccharides (hyaluronic acid or stachyose) was identified in the ESI-MS spectra. Subjecting the complexes to UVPD resulted in extensive fragmentation of each peptide, as evidenced by the sequence maps that display the peptide backbone sites cleaved by UVPD, as shown in Figures 5A, B, and S9–S11. The holo fragment ions of PR-39 and Tur1A (*i.e*., fragment ions that retain the entire tetrasaccharide) were categorized as N- or C-terminal fragments originating from each backbone cleavage site (numbered along the *x*-axis from the N-terminus to the C-terminus) and are shown in Figure 5A and B for the hyaluronic acid complexes (i.e., PR-39•Hya or Tur1A•Hya). The overlapping N- and C-terminal holo ions bracketed the region of each peptide involved in interacting with the hyaluronic acid. We found that PR-39 interacted with hyaluronic acid within a short, highly basic N-terminus region from Arg1 to Arg3, notably different from Tur1A, which interacts with hyaluronic acid over a larger region spanning Arg6 to Pro19. The interaction region between Tur1A and hyaluronic acid was greater than the interaction region observed for Bac7 (1–35)•hyaluronic acid, the latter spanning Arg6 to Arg9 (Figure S10B). However, for stachyose, the interaction region of PR-39 relocated to residues spanning Pro15 to Pro24 (Figure S11A) and the interaction region of Bac7 (1–35) relocated to Arg6 to Arg9 (Figure S10B), whereas Tur1A displayed a similar interaction region from Pro7 to Pro19 (Figure S11B). These variations suggest that PR-39 and Bac7 (1–35) engage in different binding interactions for negatively charged or neutral tetrasaccharides.

**Figure 5:**
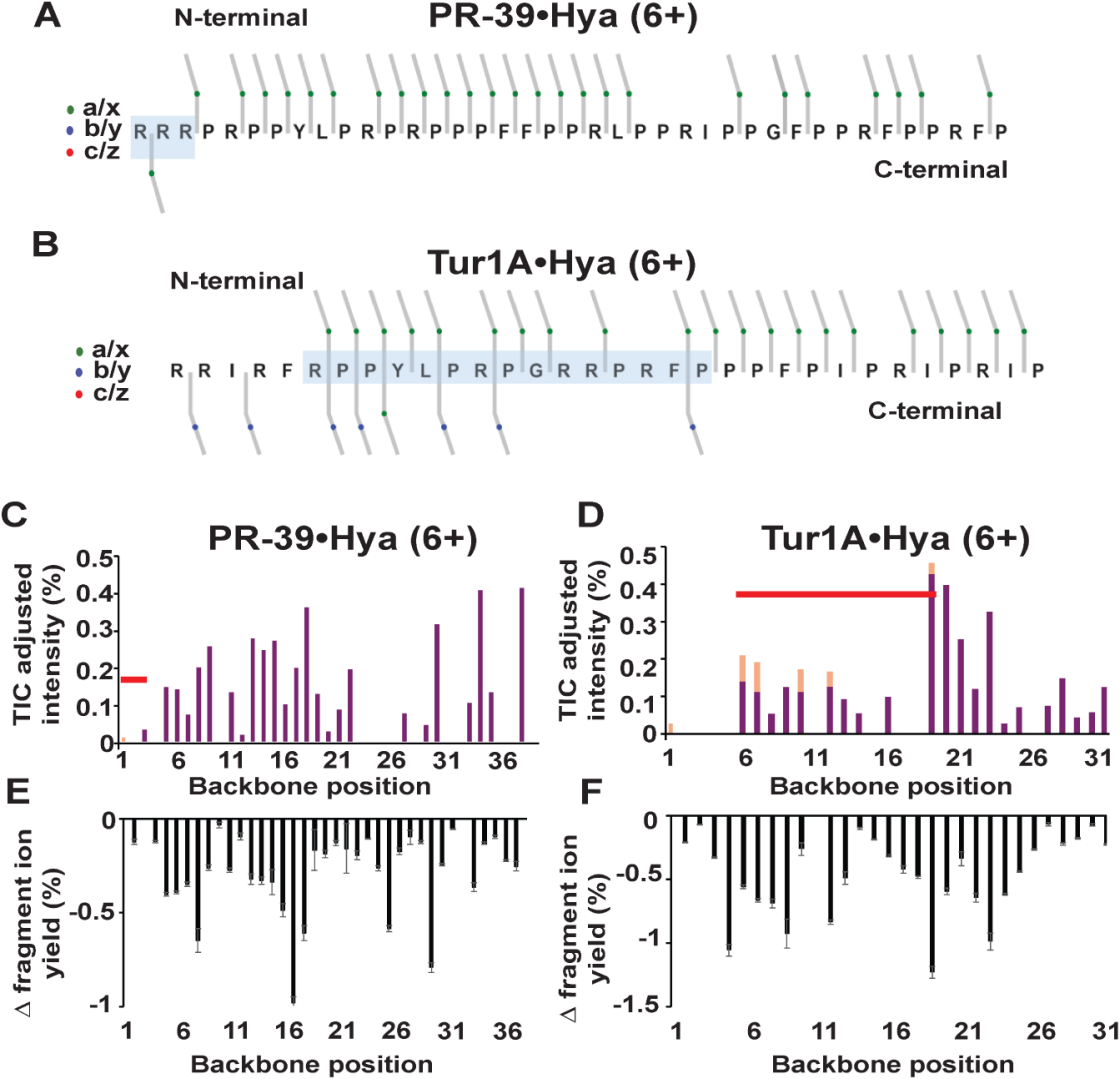
Tur1A displays increased interactions with hyaluronic acid. The figures show the holo fragment ion plots and change in fragmentation graphs based on UVPD-MS of the PR-39•Hya (6+) and Tur1A•Hya (6+) complexes. Figures 5A and 5B show holo ion fragment plots for PR-39•Hya (6+) and Tur1A•Hya (6+) complexes, respectively, with TIC-adjusted intensities of fragment ions originating from each cleavage site of the peptide backbone. The red horizontal bars on each graph indicate the hyaluronic acid binding region determined for each peptide. The change in yield of a/x fragment ions produced upon UVPD of PR-39•Hya (6+) and Tur1A•Hya (6+) complexes (summed abundances of apo and holo fragment ions) relative to a/x ions produced by the corresponding apo peptides PR-39 (6+) or Tur1A (6+) (without hyaluronic acid) is shown in Figure 5C and Figure 5D, respectively. The sequence maps for each peptide generated by UVPD are shown in Figures 5E and 5F, respectively. Graphs were generated from triplicate UVPD experiments, with standard deviations shown as error bars.

Differential fragmentation graphs reveal the variations in fragmentation efficiency when comparing the yields of *a/x* fragment ions generated by peptide•hyaluronic acid complexes (Figures 5C,D and S10D) or peptide•stachyose complexes (Figures S10C, S11C,D) relative to the corresponding apo peptides. In general, these graphs show broad suppression of fragmentation of peptide•hyaluronic acid and peptide•stachyose complexes relative to the apo peptides (evidenced as negative values on the fragment yield graphs), indicating structural stabilization and a decreased release of fragment ions. Conversely, positive values on these graphs indicate an increase in fragmentation of the peptide-tetrasaccharide complexes relative to the apo peptides, indicative of structural destabilization (i.e., enhanced fragmentation of the peptide when bound to a tetrasaccharide compared to the apo peptide). Enhanced fragmentation of the peptide-tetrasaccharide complexes was not observed for PR-39 or Tur1A when bound to hyaluronic acid and was observed for only a few backbone sites for the stachyose complexes, limiting insight derived from these minimal variations.

## Discussion

*K. pneumoniae* is a major MDR pathogen responsible for severe hospital- (cKp) and community-acquired (hvKp) infections.^56–58^ The emergence of strains combining hypervirulence and antibiotic resistance presents a major challenge for conventional therapeutics.^8,59^ PrAMPs have gained attention as promising alternatives due to their nonlytic mode of action, proteolytic stability, and ability to target intracellular bacterial processes.^60^ We previously demonstrated that the mammalian PrAMP Bac7 (1–35) exhibits potent antimicrobial activity against *K. pneumoniae*, along with the ability to aggregate with cell-associated polysaccharides and eradicate biofilms.^26^

In this study, we expanded our investigation to PrAMPs from diverse natural sources and identified that the insect peptide apidaecin Cd3^+^ and the mammalian peptides Tur1A and PR-39 exhibit comparable antimicrobial properties to Bac7 (1–35). PrAMP uptake is typically mediated by the inner membrane SbmA transporter.^61^ However, the partial retention of activity observed for PR-39 and Tur1A in an SbmA-deficient strain suggests the involvement of transporter-independent cell entry. Furthermore, we found peptides with the greatest antimicrobial activity were able to decrease cell viability within preformed biofilms and decrease the matrix height. Further analysis into potential membrane interaction of PrAMPs showed both PR-39 and Tur1A were nonlytic but strongly depolarized *K. pneumoniae* membrane, while apidaecin Cd3+ induced minimal depolarization. Peptide-induced membrane depolarization disrupts the electrochemical gradient across the bacterial cell membrane, impairing essential cellular processes such as ATP synthesis, ion transport, and maintenance of cellular homeostasis.^48^ Although select PrAMPs can bypass the SbmA transporter through direct membrane penetration, these highly charged PrAMPs are sensitive to colistin-resistant LPS modifications. We found that Bac7 (1–35) is taken into the cytosol of colistin-resistant MKP103 greater than PR-39 yet it is equally effective at killing this strain. Intriguingly, the transporter-dependent apidaecin Cd3+ displays the lowest MBCs toward colistin-resistant MKP103. This indicates sustained depolarization of the membrane may contribute to killing, although this hypothesis needs future testing to validate.

Our study expands our understanding of PrAMP diversity and function against *K. pneumoniae*, a pathogen of high clinical concern. It provides valuable insight into how structural features drive peptide activity and membrane interactions. The differential activity among the three active peptides PR-39, Tur1A, and apidaecin Cd3^+^ suggests that variations in amino acid composition critically influence how PrAMPs navigate bacterial barriers such as *K. pneumoniae* extracellular capsule and LPS to engage their intracellular targets. These findings highlight the need for further investigation into the molecular determinants that govern PrAMP mechanisms of action and how this knowledge can be applied to design more effective treatment strategies against multidrug-resistant *K. pneumoniae* infections.

## Methods

### Bacterial Strains and Peptides

The bacterial strains utilized in this study are listed in Table S1. The plasmid pMF230, containing the Green Fluorescent Protein (GFP) reporter gene, was obtained from Addgene (pMF230 deposited by Michael Franklin; Addgene plasmid #62546; http://n2t.net/addgene:62546; RRID:Addgene 62546) and introduced into *K. pneumoniae* NTUH-K2044 via conjugation, following established protocols.^62^ All PrAMPs used in this study were synthesized by Novopro, and polymyxin B sulfate was purchased from TCI Chemicals. PR-39-BODIPY (493/503) (MW 5149.98) and Bac7 (1–35)-BODIPY (493/503) (MW 4637.44) were synthesized by Novopro using Lys-BODIPY (493/503) to label the C-terminal and retain the N-terminal activity of the peptides. Peptides were dissolved in ultrapure water at a concentration of 10 mg mL^–1^ and stored at −20 °C.

### Antibacterial Assays

MIC assays were conducted in 96-well plates using an adapted broth dilution method.^32^ Bacteria were grown overnight, synchronized to the exponential phase, and standardized to an optical density of 600 nm (OD_600_) of 0.001 (1 × 10^5^ CFU mL^–1^) in MHB1. Peptides were prepared using 2-fold serial dilutions, ranging from 128 μg mL^–1^ to 0.25 μg mL^–1^ (∼70 μmol L^–1^ to ∼0.05 μmol L^–1^), in a solution of 0.2% BSA and 0.01% acetic acid.^32^ Standardized cultures were added to the 96-well plates, which were then incubated at 37 °C for 24 h. OD_600_ readings were taken using a plate reader to determine MIC values, defined as the lowest peptide concentration that inhibited bacterial growth after 24 h of incubation at 37 °C. All MICs were established by triplicate, confirming the results. For MBC assays, the same procedure for MIC was done, followed by spot plating 5 μL of the 96-well plate onto Luria–Bertani (LB) agar plates and incubating at 37 °C for the selected time points.

### Peptide Sequence Alignment

The phylogenetic relationships among the studied peptides were analyzed using MEGA version 11 software, following previously described methods.^63^ Peptide sequences were obtained from Welch et al.^29^ and saved in FASTA format. Multiple sequence alignment of the amino acid sequences was performed using the MAFFT algorithm implemented in MEGA version 11 with default parameters. The resulting amino acid alignment was used to infer phylogenetic relationships using the Neighbor-Joining method. Evolutionary distances were calculated by using the Poisson correction substitution model. The phylogenetic tree was visualized and edited in MEGA.

### Cell-Associated Polysaccharides Extraction, Purification, and Quantification

The cell-associated polysaccharides were extracted using the hot phenol method.^64^ Briefly, an overnight culture of 500 mL of *K. pneumoniae* MKP103 was centrifuged at 3434 × g, 4 °C, for 15 min. The pellet was washed once with water and resuspended in 50 mL of water. The sample was incubated at 68 °C for 2 min, followed by the addition of 500 μL of phenol and incubation for 30 min at 68 °C. After incubation, 500 μL of chloroform was added, and the samples were centrifuged at 32300 × g for 5 min to separate the aqueous layer. To precipitate polysaccharides, three volumes of ethanol were added, and the mixture was incubated overnight at −20 °C. The sample was centrifuged at 32300 × g for 30 min, and ethanol was removed. The pellet was resuspended in 500 μL of water and dialyzed overnight against water using a 100 Da cutoff dialysis membrane. Samples were recovered from the dialysis membrane, dried in an Eppendorf Vacufuge Plus, and resuspended at 10 mg mL^–1^ in 0.8% NaCl, 0.05% NaN_3_, and 0.1 M Tris-HCl (pH 7). The solution was treated with DNase II (50 μg mL^–1^) and RNase A (50 μg mL^–1^) for 18 h at 37 °C, followed by Proteinase K digestion (50 μg mL^–1^) for 1 h at 55 °C and an additional 24 h at room temperature. Polysaccharides were then precipitated with five volumes of methanol containing 1% (v/v) saturated sodium acetate and incubated overnight at −20 °C. Finally, the pellet was resuspended in molecular-grade water and ultracentrifuged at 105,000 × g for 20 h at 4 °C and repeated twice to remove impurities. Polysaccharide content was quantified by using the uronic acid assay.

### Peptide–Polysaccharide Aggregation Assay

Peptide aggregation with extracellular polysaccharides was performed using 100 μg mL^–1^ of extracted cell-associated polysaccharides diluted in 10 mmol L^–1^ potassium phosphate buffer, mixed with 100 μmol L^–1^ of each peptide to a final volume of 200 μL. Samples were centrifuged at 21300 × g for 15 min, after which the supernatant was discarded and pellets were resuspended in 10 μL of sterile water. A control without peptide was added to ensure to account for polysaccharide retention in the microcentrifuge tubes in the absence of peptide. To evaluate cell-associated polysaccharides within the aggregates, resuspended pellets were resolved on 4–8% bis-tris SDS-PAGE gels and stained with Alcian blue, as described previously.^65^ Each assay was performed in triplicate. Gel band densities were quantified using ImageJ, with control values subtracted as the background, and results were plotted for analysis.

### Biofilm Disruption Analyses

#### Enumeration of Cell Viability with and without PrAMPs

*K. pneumoniae* NTUH-K2044 strain, constitutively expressing GFP, was cultured overnight in LB broth at 37 °C with shaking with 800 μmol L^–1^ carbenicillin to retain the plasmid. Overnight cultures were diluted to an OD_600_ of 0.5 in biofilm media (tryptic soy broth supplemented with 0.5% glucose). One milliliter aliquots of the standardized culture were seeded into 35 mm culture dishes and incubated statically at 37 °C for 24 h to allow biofilm formation. Peptides were prepared by resuspending them in 1 mL of BSA buffer (0.2% BSA and 0.01% acetic acid) and mixed with 7 mL of MHB1 to achieve a final concentration of 64 μg mL^–1^ (∼30 μmol L^–1^). The supernatant was removed from the preformed biofilms and replaced with the peptide solution, which was then incubated statically at 37 °C for 24 h. Following treatment, the culture supernatant containing dispersed biofilm cells was collected and serially diluted using 2-fold dilutions in 1× PBS supplemented with 2% saponin. The attached biofilms were gently washed with 1 mL of 1× PBS to remove nonadherent cells, followed by the addition of 1 mL of 1× PBS containing 2% saponin for biofilm disruption. The resulting biofilm suspensions were then serially diluted using 2-fold dilutions in 1× PBS with 2% saponin. Serially diluted dispersed (supernatant) and biofilm-associated cell populations were then plated on LB agar plates for enumeration of viable cells. All experiments were conducted in triplicate, with error shown as ±SEM and significance calculated using one-way ANOVA with Dunnett’s correction for multiple comparisons.

#### Biofilm Microscopy Imaging

*K. pneumoniae* NTUH-K2044 strain, constitutively expressing GFP, was cultured overnight in LB broth at 37 °C with shaking with 800 μmol L^–1^ carbenicillin to retain the plasmid. Overnight cultures were diluted to an OD_600_ of 0.5 in biofilm media (tryptic soy broth supplemented with 0.5% glucose). A 1 mL aliquot of the standardized culture was seeded into 35 mm Matsunami glass-bottom culture dishes (VWR) with a glass thickness of no. 1.5 (0.16–0.19 mm) and incubated statically at 37 °C for 24 h to allow biofilm formation. Peptides were prepared by resuspending them in 1 mL of BSA buffer (0.2% BSA and 0.01% acetic acid) and mixed with 7 mL of MHB1 to achieve a final concentration of 64 μg mL^–1^ (∼30 μmol L^–1^). The supernatant was removed from the preformed biofilms and replaced with the peptide solution, which was then incubated statically at 37 °C for 24 h. Following treatment, the supernatant was removed, and the biofilms were washed with 1 mL of 1× PBS. The biofilm polysaccharide matrix was stained using Texas Red-conjugated concanavalin A (1 mmol L^–1^, Invitrogen) resuspended in 0.1 M sodium bicarbonate. The dishes were gently rocked in the dark for 5 min on a gel rocker. Excess dye was removed by washing the biofilms with 1 mL of 1× PBS. The stained biofilms were imaged using a Zeiss LSM 710 confocal microscope equipped with a 63× oil objective and 488 and 543 nm laser channels. The Z-stack imaging was employed to generate 3D renderings of the biofilms.

### Inner Membrane Permeability Assay

To assess inner membrane leakage induced by PrAMPs, we adapted the previously described method.^66^ Exponentially growing cultures were centrifuged at 3434 × g at 23 °C for 10 min, followed by a single wash with 10 mmol L^–1^ sodium phosphate buffer (pH 7.4). The resulting pellet was resuspended in 5 mL of the same buffer and standardized to an OD_600_ value of 0.1. ONPG was added to the standardized cultures at a final concentration of 1.5 mmol/L^–1^. Aliquots of 50 μL were transferred into a black, clear-bottom 96-well plate containing the desired peptide concentrations. The production of o-nitrophenol, indicative of membrane leakage, was monitored at an absorbance of 410 nm every 3 min for 45 min. The assay was conducted in triplicate, and data are presented as mean ± SEM. Statistical significance was determined using an ordinary one-way ANOVA test.

### ATP Bioluminescence Assay

Cellular ATP leakage was assessed using the standard protocol^67^ with slight modifications. Overnight cultures were grown to the exponential phase and standardized to an OD_600_ of 0.5 in 2 mL of MHB1. The standardized cultures were then treated with peptides for 1 h, followed by centrifugation at 3434 × g for 10 min. The resulting supernatants were filtered using 0.22 μm syringe filters. ATP levels in the supernatant were quantified using the CellTiter-Glo 2.0 assay (Promega). 100 μL of supernatant was mixed with an equal volume of CellTiter-Glo 2.0 reagent in a black 96-well plate and incubated at 37 °C in a plate reader for 10 min. Luminescence was read immediately after incubation. The assay was done in triplicate, with data presented as mean ± SEM.

### Inner Membrane Depolarization Assay

Inner membrane depolarization was assessed by tracking the fluorescence kinetics of the (3,3′-dipropylthiadicarbocyanine iodide) DiSC_3_(5) dye (ThermoFisher), following the protocol by Lemonche et al.^49^ with modifications. Mid-log-phase bacterial cultures were centrifuged at 3434 × g for 10 min, the supernatant was removed, and the pellet was resuspended in 20 mL of Buffer A (5 mM HEPES, 5 mM glucose, pH 7.2). This washing step was repeated twice. The final pellet was resuspended in 5 mL of Buffer A, then standardized to an OD_600_ of 0.1 in a solution containing Buffer A and 100 mM KCl. DiSC_3_(5) dye was added to a final concentration of 2 μmol L^–1^. Aliquots of 50 μL were transferred into a black, clear-bottom 96-well plate. The plate was incubated at 37 °C, and fluorescence (excitation = 622 nm, emission = 670 nm) was measured every 2 min for 30 min to establish a baseline. After fluorescence stabilization, the plate was removed, and 2-fold serially diluted peptides were added to triplicate wells. The plate was immediately returned to the reader, and fluorescence measurements continued every 2 min for another 30 min. At the end of the fluorescence readings, 5 μL from each well was spotted onto an LB agar plate to evaluate bacterial viability after 30 min of peptide incubation. The 96-well plate was then incubated statically at 37 °C. Additional 5 μL aliquots were taken at 1, 2, 4, and 24 h time points and spotted onto LB agar plates to monitor the kinetics of growth inhibition.

### Flow Cytometry with BODIPY-Tagged PrAMPs

This procedure was adapted from previously described methods.^47^ To assess changes in PrAMP uptake, cells were treated with C-terminal-tagged BODIPY PrAMPs and analyzed by flow cytometry. MKP103 parental isolate, MKP103 Δ*sbmA*, and MKP103 Δ*phoP* were treated with 4 μmol L^–1^ Bac7 (1–35)-BODIPY or PR-39-BODIPY in MHB1 for 30 min, 4 h, and 24 h. Following incubation, cells were harvested by centrifugation at 3434 × g for 10 min and resuspended in 1× PBS to remove residual peptide. BacLight Red Bacterial Stain (Invitrogen) was added to a final concentration of 200 μmol L^–1^, and samples were incubated at room temperature for 15 min. Flow cytometric analysis was performed using a CytoFLEX S flow cytometer, with fluorescence detected using the FITC (green) and APC (red) channels. The samples were run in triplicate, and data were analyzed using FlowJo software (v10, BD Biosciences). Debris were excluded using FSC-A and SSC-A gating, while doublets were excluded using FSC-A and FSC-H. The untreated control (no peptide) with parental MKP103 was used to establish gating parameters for background green fluorescence and cell staining, to apply to all other samples. Representative pseudocolor dot plots are shown in Figure 4 and the total count of the quadrants from triplicate samples was exported as an Excel spreadsheet to generate the graphs shown in Figure S8 with error reported as ±SEM.

### ESI-Mass Spectrometry and Ultraviolet Photodissociation

ESI mass spectrometry was performed on a Thermo Scientific Orbitrap Eclipse Tribrid mass spectrometer modified with a 193 nm laser for UVPD. Ten μmol L^–1^ peptide was prepared in 5 m mol L^–1^ NH_4_OAc aqueous solution. For peptide•tetrasaccharide complexes, 40 μmol L^–1^ stachyose or 160 μmol L^–1^ hyaluronic acid (amsbio: AMS.HA04) was added, and the mixture was incubated with the peptide for 5 min prior to direct infusion ESI-MS analysis. Samples were infused via static nanoelectrospray ionization (ESI) using AuPd-coated borosilicate glass emitters pulled in-house, with 900–1200 V ESI voltage applied. UVPD spectra were collected for the most abundant charge states (5+) using 1 laser pulse set to 2 mJ. An automatic gain control (AGC) target of 1000% was used, along with 60K resolution and averaging 100 scans. Spectra were deconvoluted using Xtract and analyzed using MS-TAFI.^68^ N-terminal fragment ions (*a,a+1,b,c)* and C-terminal fragment ions (*x,x+1,y,y-1,y-2,z*) were identified. For differential fragmentation plots, differences in total ion current (TIC)-adjusted intensity of a/x fragments between stachyose-bound and unbound peptides were calculated, summing both apo and holo fragments.

## Supplementary Material



## Notes

Figure S1: Minimum bactericidal concentration (MBC) of peptides in phosphate-buffered saline; Figure S2: Cell-attached polysaccharide aggregation and band density quantification; Figure S3: Confocal z-stack imaging of PrAMPs; Figure S4: Spot plating after the DiSC3 assay; Figure S5: PrAMP membrane depolarization of clinical isolates, MKP103, and the MKP103 ΔsbmA transporter mutant; Figure S6: Spot plating for MBC assessment with MKP103 and bacterial mutants; Figure S7: MBC after PrAMP treatment; Figure S8: Quantification of BODIPY-tagged peptide uptake by flow cytometry; Figure S9: Apo fragment ion plots based on UVPD of peptide•tetrasaccharide complexes; Figure S10: TIC-adjusted intensity of the holo fragment ions (tetrasaccharide-retaining ions) originating from each backbone cleavage position is plotted along the peptide sequence from the N-terminus to the C-terminus for UVPD of: (A) Bac7(1–35)•stachyose (5+) and (B) Bac7(1–35)•hyaluronic acid (6+); Figure S11: TIC-adjusted intensities of the holo fragment ions (retaining stachyose) originating from each backbone cleavage position are plotted along the peptide sequence from the N-terminus to the C-terminus for UVPD of: (A) PR-39•stachyose (5+) and (B) Tur1A•stachyose (5+) complexes; and Table S1: Bacterial isolates used in this study. Supplemental References (PDF)
