# Supplementary material for: Characterization of Proline-Rich Antimicrobial Peptides with SbmA Transporter-Dependent and Independent Antimicrobial Activity toward Klebsiella pneumoniae
Source: ACS Infect Dis. 2026 Apr 20;12(5):1751–64. doi: 10.1021/acsinfecdis.6c00142 (PMC13162253; doi:10.1021/acsinfecdis.6c00142)
Supplement: Supplementary file 1 [file id6c00142_si_001.pdf]

Supporting Information File

Characterization of Proline-rich Antimicrobial Peptides with SbmA Transporter Dependent and Independent Antimicrobial Activity towards *Klebsiella pneumoniae*

Ridhwana M. Appiah[1], Robert L. Beckman IV[1], Christina M. DeBarro[1], Hanlin Ren [2], Jennifer S. Brodbelt [2], Renee M. Fleeman[1*]

[1] Division of Immunity and Pathogenesis, Burnett School of Biomedical Sciences, College of Medicine, University of Central Florida. Orlando, FL 32827

[2] Department of Chemistry, The University of Texas at Austin. Austin, TX 78712

*Correspondence: Renee.Fleeman@ucf.edu

ORCID account = https://orcid.org/0000-0001-7103-461X

Keywords: *Klebsiella pneumoniae*, PrAMPs, extracellular polysaccharide, Biofilms

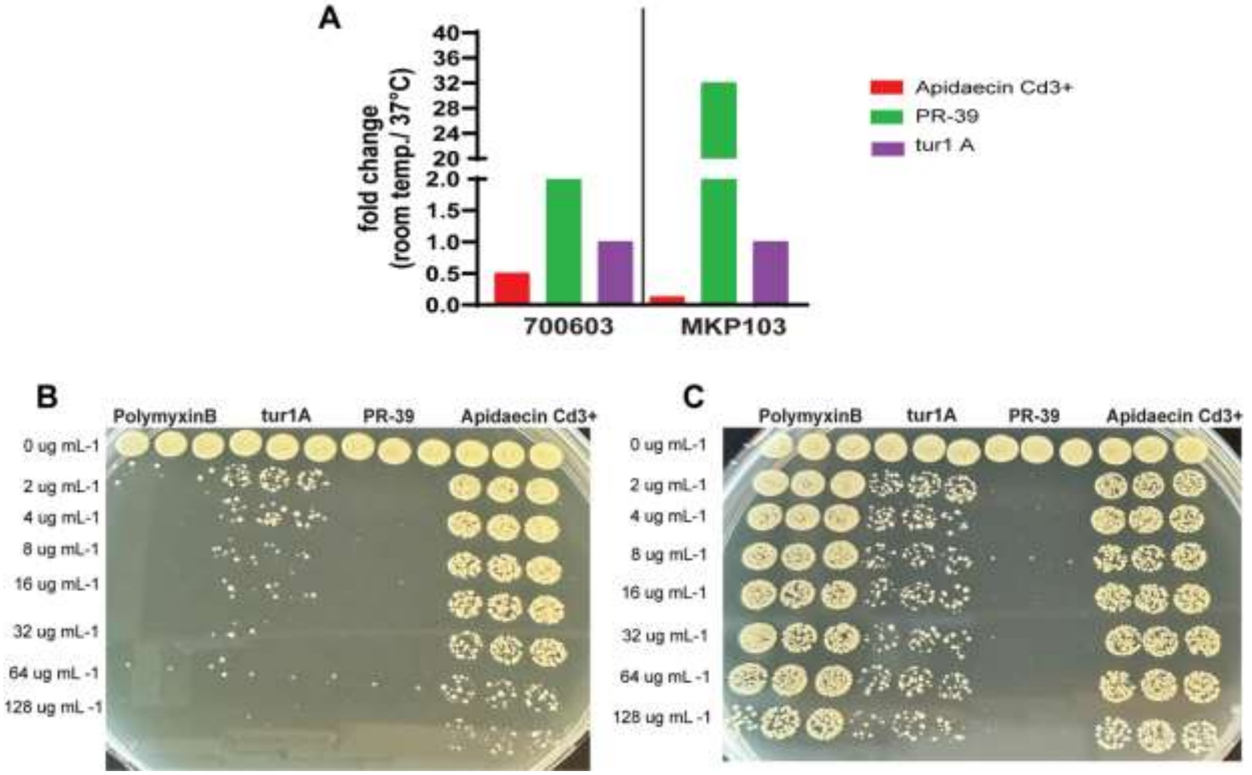

Figure S1. Minimum bactericidal concentration (MBC) of peptides in phosphate buffered saline. The figures show PrAMP killing at room temperature in Mueller Hinton broth and 1X PBS. Peptides were incubated in triplicate with ATCC 700603 and MKP103 in MHB for 48 hours at room temperature and optical density was used to determine MIC values to quantify fold change between room temperature and 37 °C MICs (**Figure S1A**). Peptides were incubated in triplicate in 96-well plates for 24 hours in 1X PBS at room temperature before spot plating on LB agar. **Figure S1B** and **S1C** shows the spot plating of peptides against ATCC 700603 and MKP103, respectively. MBC was determined by concentration where no colonies grow.

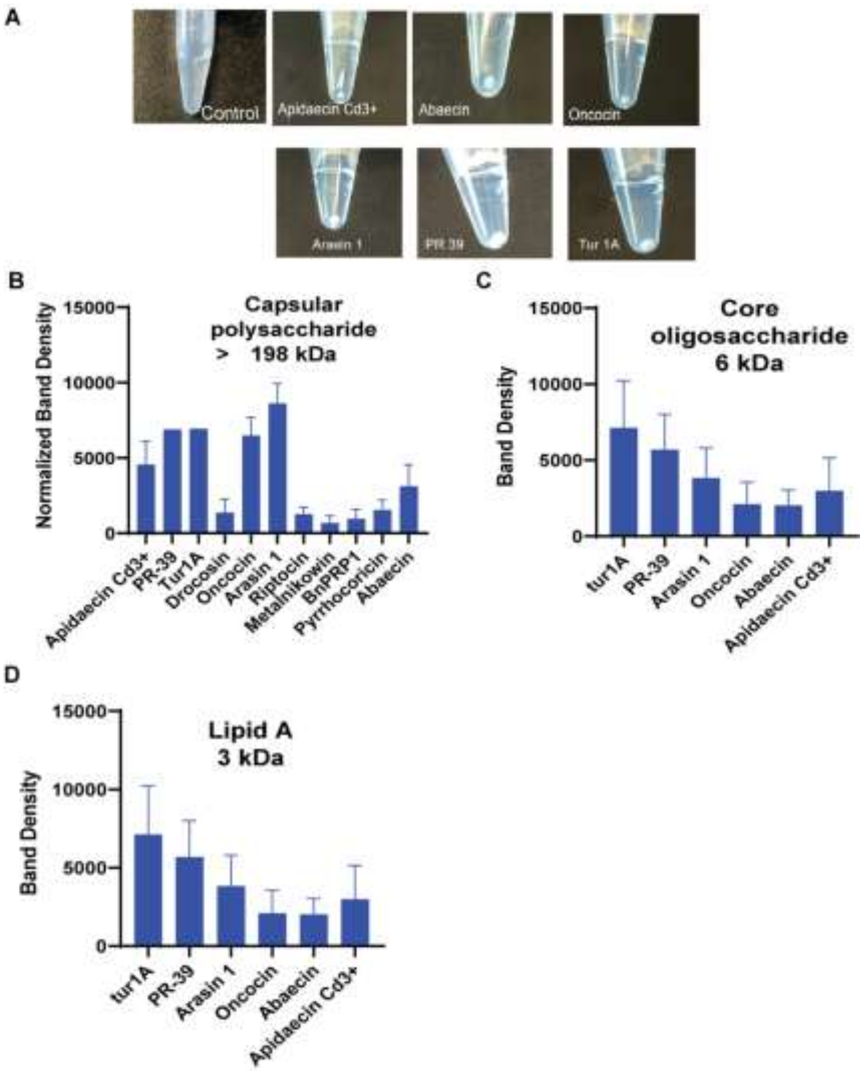

Figure S2. Cell-attached polysaccharide aggregation and band density quantification. The figures show the aggregates that were used for the SDS page analysis and the ImageJ band density assessment to quantify CPS and LPS. **Figure S2A** shows the peptide•polysaccharide aggregate following centrifugation of PrAMPs (100 µmol L^{-1}) with EPS (100 µg mL^{-1}) for 15 minutes. **Figures S2B-S2D** show the ImageJ quantification of the SDS page band density of CPS, LPS core, and LPS lipid A, respectively. The CPS was normalized by subtracting the control band density, while LPS bands show band density without normalization. Triplicate aggregation and SDS page gels were imaged and quantified with error shown at ±SEM and representative aggregates shown for **Figure S2A**.

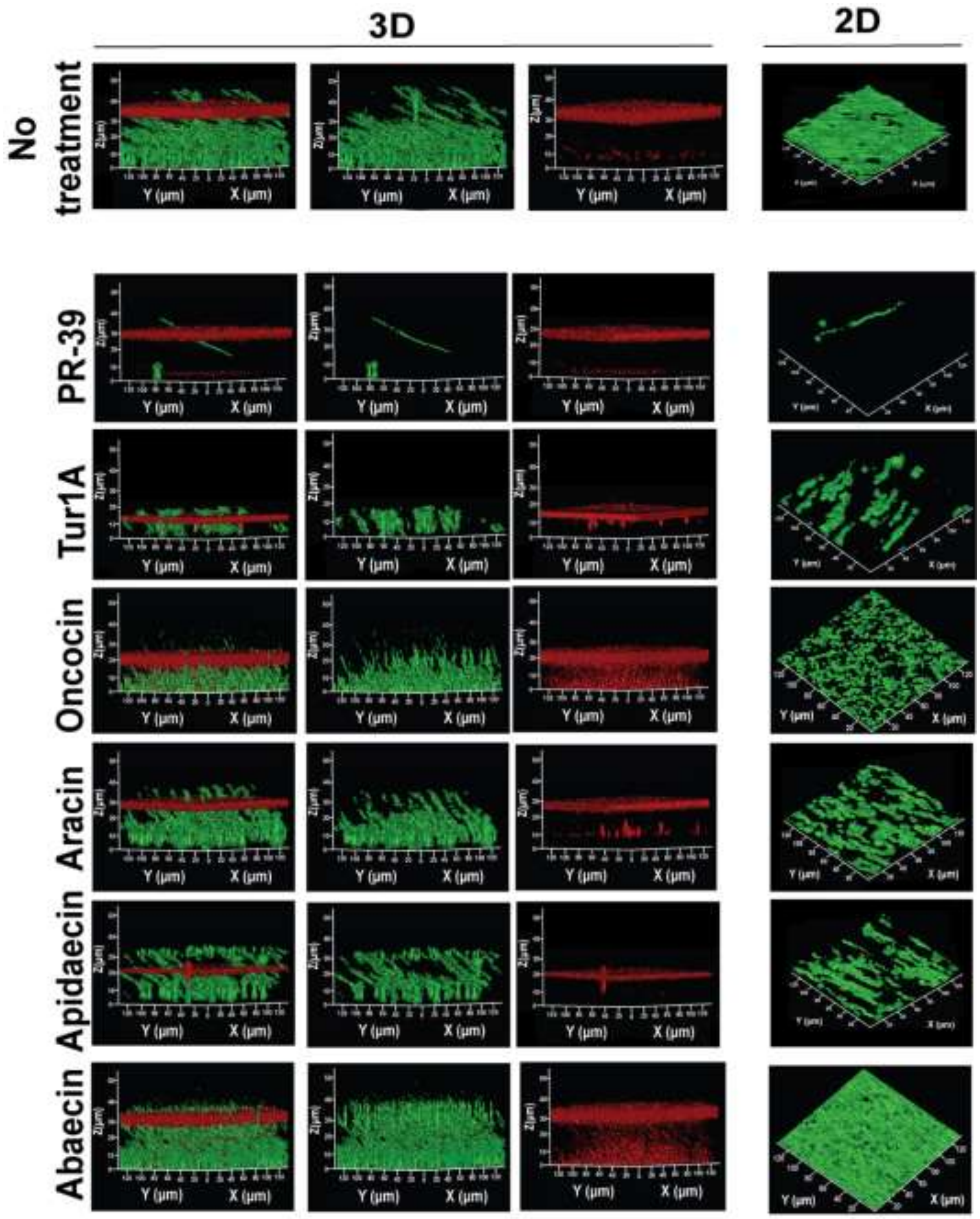

Figure S3. Confocal z-stack imaging of PrAMPs. The figures show 3D rendering and 2D images of confocal z-stack imaging of *K. pneumoniae* NTUH-K2044 constitutively expressing GFP with concanavalin A-Texas Red stain for polysaccharides. The biofilm imaging was performed in triplicate with a representative image shown.

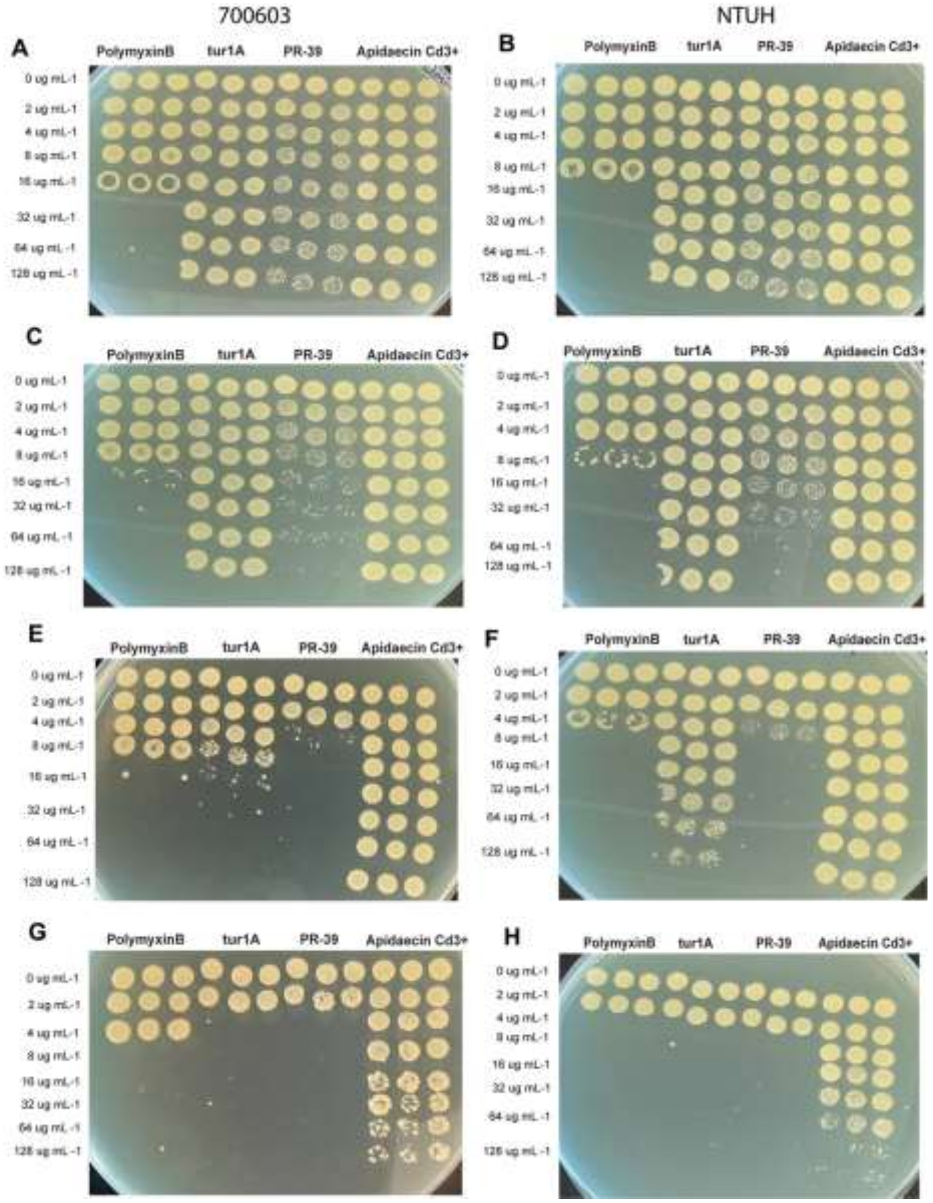

Figure S4. Spot plating after DiSC$_3$ assay. The photos show spot plating of triplicate 2-fold dilutions of peptides in 96 well plates used for the DiSC$_3$ kinetic fluorescence assay. The plating was done immediately after DiSC$_3$ assessments using ATCC 700603 and NTUH-K2044 at 30 minutes (**Figure S4A** and **S4B**), 1 hour (**Figure S4C** and **S4D**), 4 hours (**Figure S4E** and **S4F**), and 24 hours (**Figure S4G** and **S4H**).

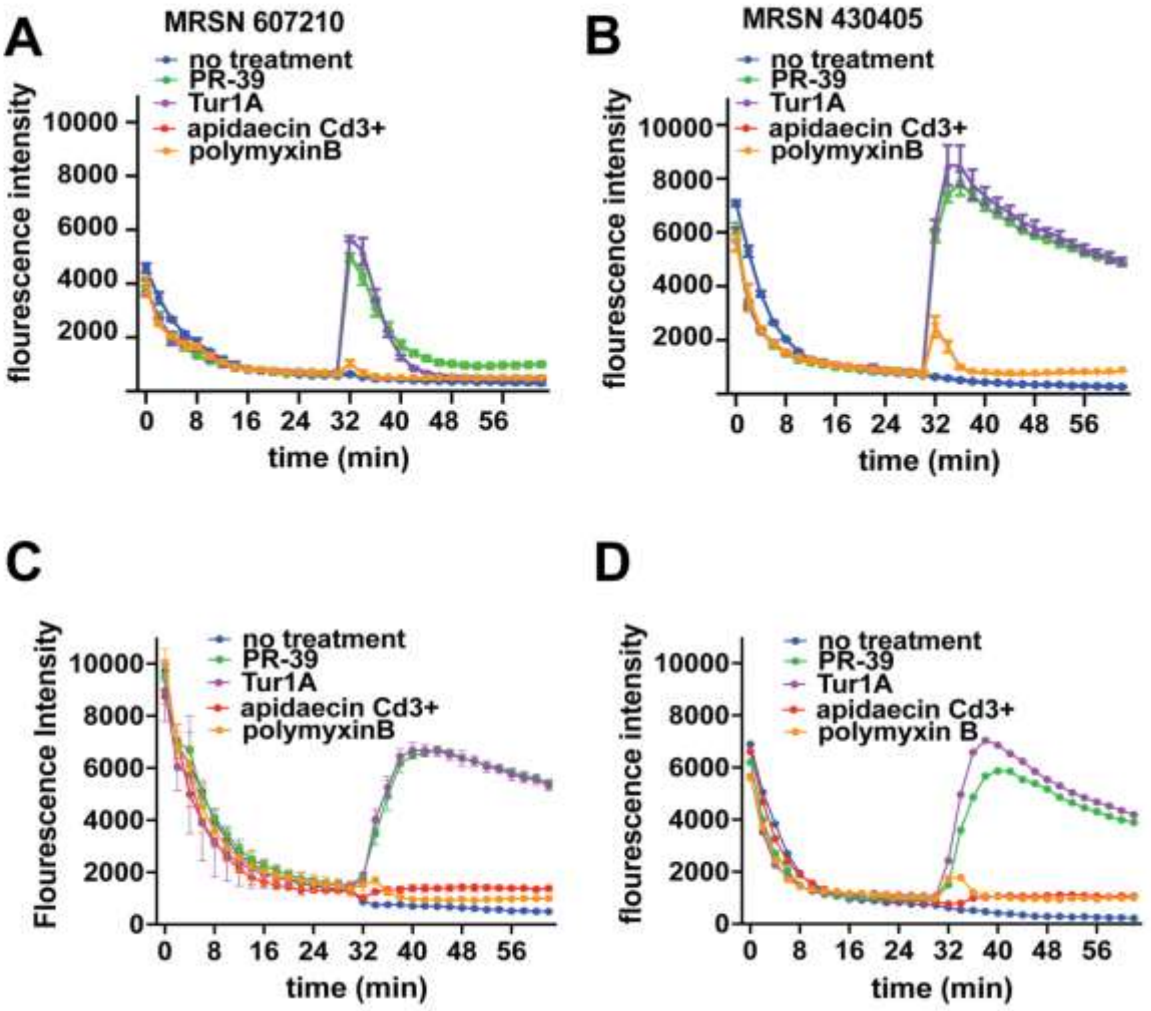

Figure S5. PrAMP membrane depolarization of clinical isolates, MKP103, and MKP103 Δ*sbmA* transporter mutant. The figure shows the membrane depolarization by PrAMPs using the $DiSC_3$ kinetic fluorescence assay. **Figure S5A** shows the 30-minute treatment with 1 µmol L^{-1} of PrAMPs following 30-minute dye quenching testing with the colistin sensitive MRSN 607210. **Figure S5B** shows the 30-minute treatment with 1 µmol L^{-1} of PrAMPs following 30-minute dye quenching testing with the colistin resistant MRSN 430405. **Figure S5C** shows the 30-minute treatment with 1 µmol L^{-1} of PrAMPs following 30-minute dye quenching testing with the colistin resistant parental lab strain MKP103. **Figure S5D** shows the 30-minute treatment with 1 µmol L^{-1} of PrAMPs following 30-minute dye quenching testing with MKP103 Δ*sbmA*. All assays were performed in triplicate with error shown at ±SEM.

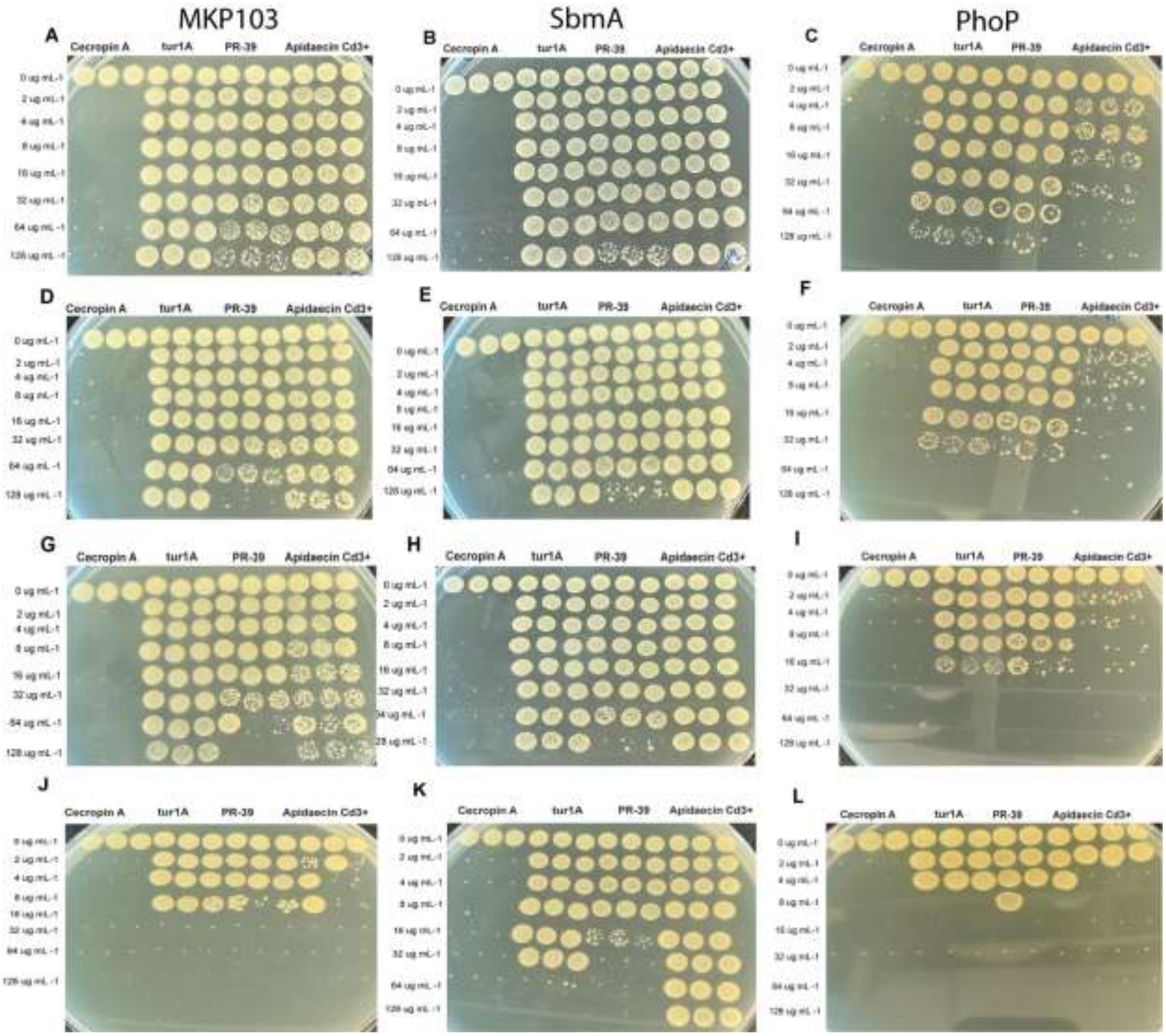

Figure S6. Spot plating for MBC assessment with MKP103 and bacterial mutants. The photos show spot plating of 2-fold dilutions of cecropin lytic peptide, Tur1A, PR-39, and apidaecin Cd3+ in 96 well plates in MHB1 over 24-hour incubation to determine MBCs. MKP103, MKP103 Δ*sbmA,* MKP103 Δ*phoP* at 30 minutes, (**Figure S6A-S6C**), 1 hours (**Figure S6D-S6F**), 2 hours (**Figure S6G-S6I**), and 24 hours (**Figure S6J-S6L**).

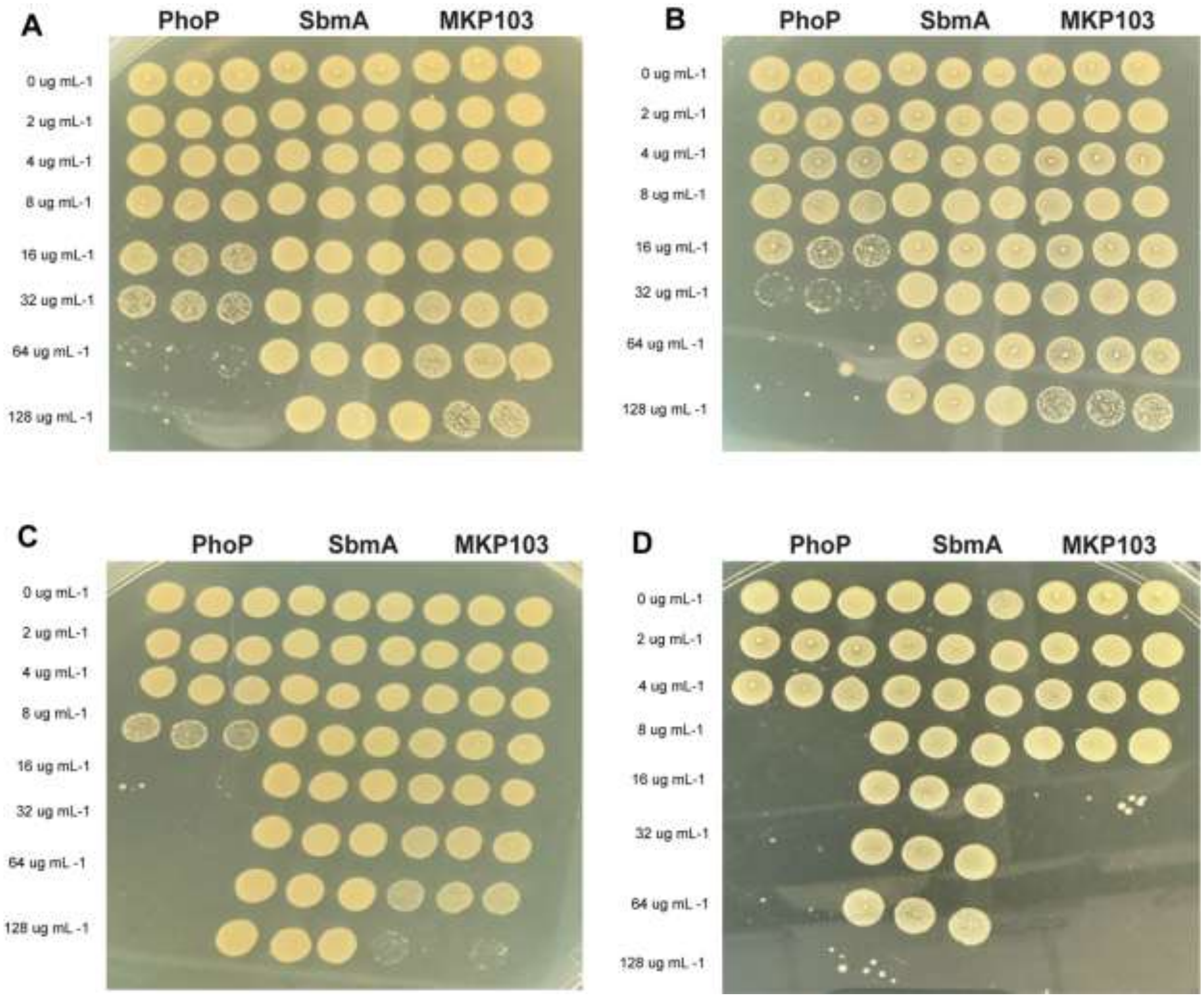

Figure S7. **MBC after PrAMP treatment.** The photos show spot plating of 2-fold dilutions of Bac7 (1-35) in 96 well plates in MHB1 over 24-hour incubation to determine MBCs. MKP103, MKP103 Δ*sbmA,* MKP103 Δ*phoP* at 30 minutes, (**Figure S7A**), 1 hours (**Figure S7B**), 2 hours (**Figure S7C**), and 24 hours (**Figure S7D**).

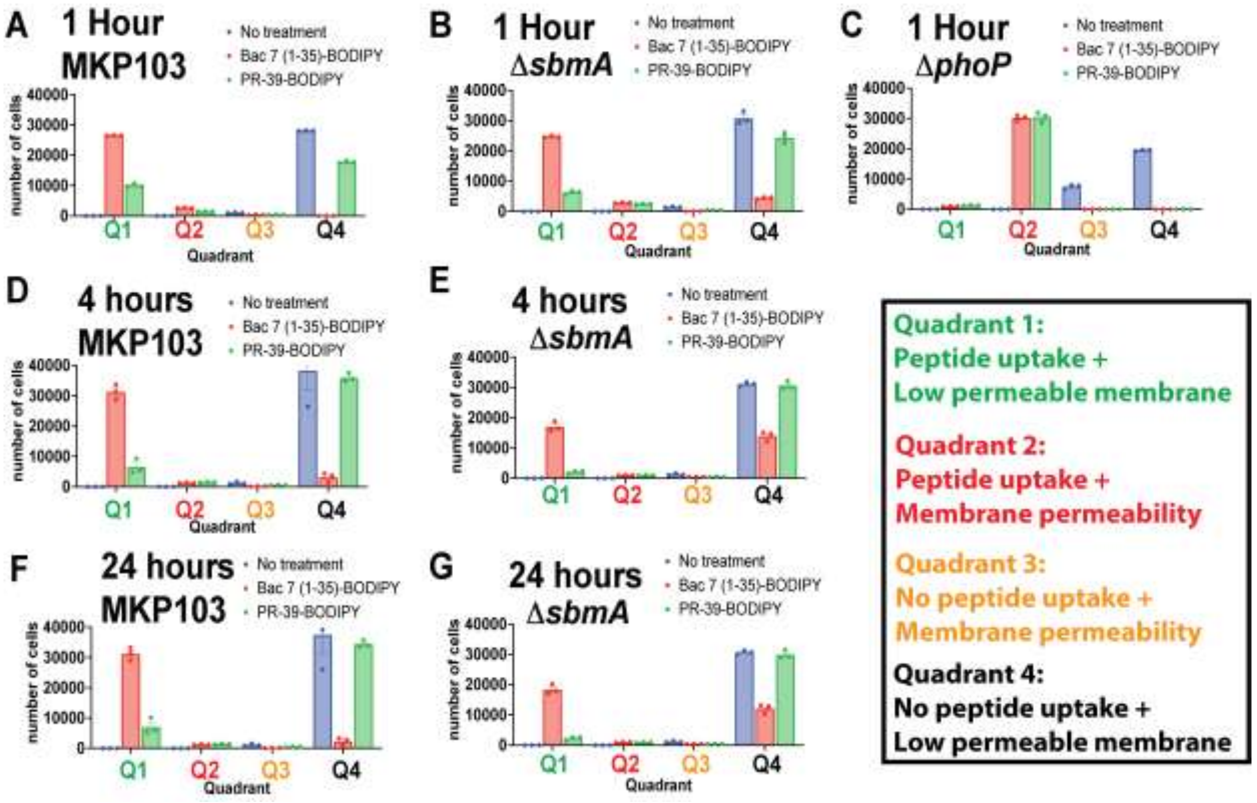

Figure S8. **Quantification of BODIPY tagged peptide uptake by flow cytometry.** The figures summarized the results of triplicate experiments of Bac7 (1-35)-BODIPY and PR-39-BODIPY peptide uptake using flow cytometry analysis quantifying the cells in the quadrants of the pseudoplot after gating. Gating was used to separate peptide uptake (Q1 and Q2) from no uptake (Q3 and Q4) in addition to increased membrane permeability (Q2 and Q3). MKP103 was tested next to *sbmA* and *phoP* mutants for 1 hour (**Figure S8A-S8C**). MKP103 was tested next to *sbmA* mutant for 4 hours (**Figure S8D** and **S8E**). MKP103 was tested next to *sbmA* mutant for 24 hours (**Figure S8F** and **S8G**). Error is graphed as ±SEM.

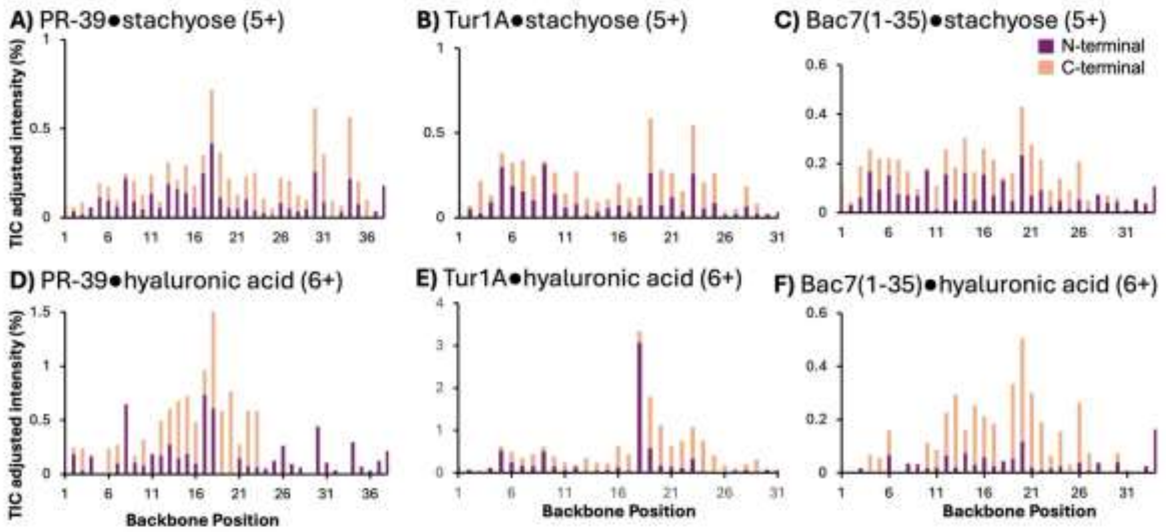

Figure 9. Apo fragment ion plots based on UVPD of peptide•tetrasaccharide complexes. The apo fragment ions originating from each backbone cleavage position are plotted along the peptide sequence from N-terminus to C-terminus. Apo fragment ion plots are shown for A) PR-39●stachyose (5+), B) Tur1A●stachyose (5+), C) Bac7(1-35)●stachyose (5+), D) PR-39●hyaluronic acid (6+), E) Tur1A●hyaluronic acid (6+), and F) Bac7(1-35)●hyaluronic acid (6+).

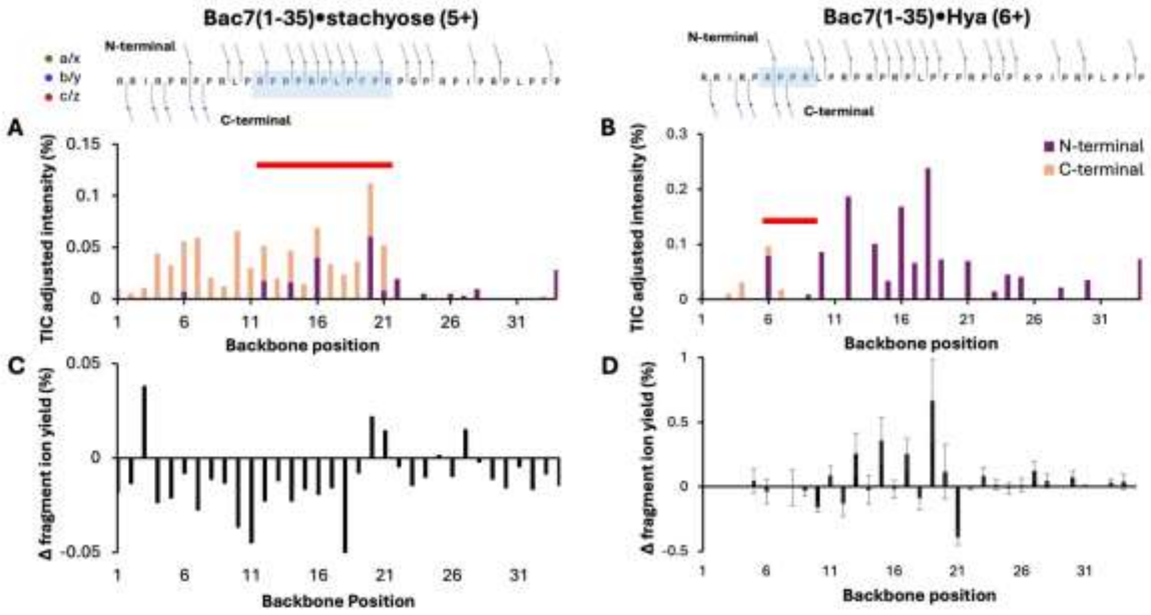

Figure S10. **TIC adjusted intensity of the holo fragment ions (tetrasaccharide-retaining ions) originating from each backbone cleavage position are plotted along the peptide sequence from N-terminus to C-terminus for UVPD of: (A) Bac7(1-35)•stachyose (5+) and (B) Bac7(1-35)•hyaluronic acid (6+)**. The red bar indicates ligand binding region determined based on holo ions. The changes in yield of *a/x* fragment ions produced upon UVPD are plotted as difference plots for (C) Bac7(1-35)•stachyose (5+) and (D) Bac7(1-35)•hyaluronic acid (6+), summing abundances of both apo and holo *a/x* fragment ions of Bac7•tetrasaccharide complexes relative to *a/x* ions produced by apo (unbound) Bac7(1-35) of the same charge states.

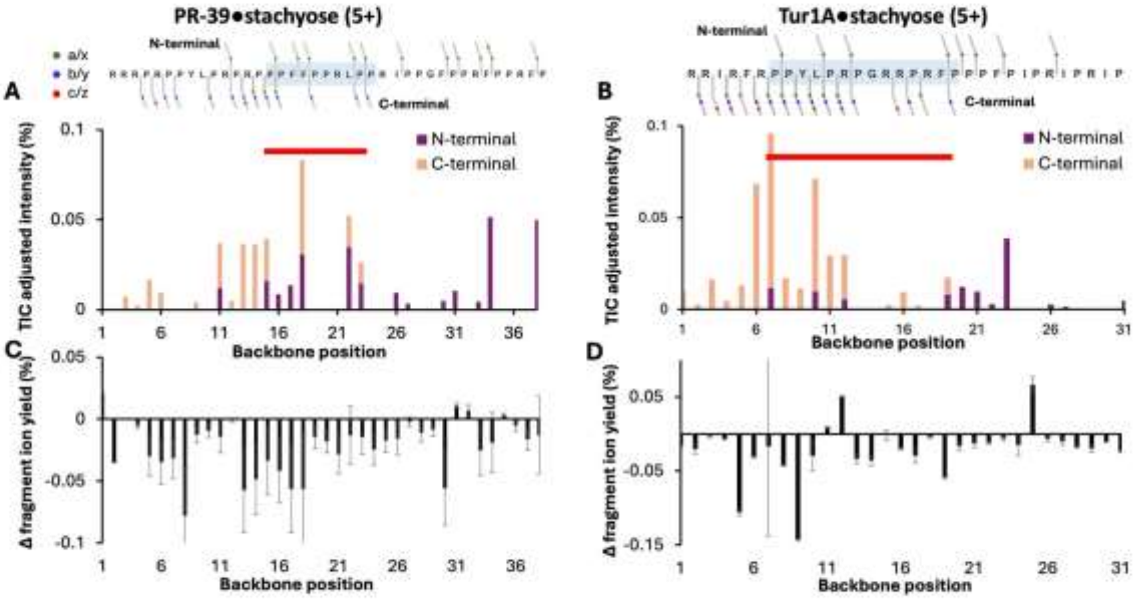

Figure S11. TIC adjusted intensities of the holo fragment ions (retaining stachyose) originating from each backbone cleavage position are plotted along the peptide sequence from N-terminus to C-terminus for UVPD of: A) PR-39•stachyose (5+) and B) Tur1A•stachoyse (5+) complexes. The sequence maps for each peptide generated by UVPD are shown at the top. The red horizontal bars on each graph indicate the stachyose binding region determined for each peptide. The changes in yields of *a/x* fragment ions produced upon UVPD of C) PR-39•stachyose (5+) and D) Tur1A•stachyose (5+) complexes (summing abundances of apo and holo *a/x* fragment ions of peptide•stachyose complexes relative to a/x ions produced by the corresponding apo peptides PR-39 or Tur1A (without stachyose, 5+) are displayed as difference plots. Graphs were generated from triplicate UVPD experiments with standard deviations shown as error bars.

Table S1: Bacterial isolates used in this study.

	Characteristics	Isolation	Reference
E. coli			
W3110	Type strain	K-12 lineage	1
A. baumannii			
5075	MDR	Tibia / Osteomyelitis	2
K. pneumoniae			
ATCC 43816	K2 capsule serotype	Pneumonia isolate	3
ATCC 13883	Type strain		4
NTUH-K2044	K1 capsule serotype		5
MKP103	ST258	NIH clinical center outbreak	6
KPNIH1-05310-803::T_{30}	SbmA deficient	Transposon mutant of MKP103	6
ATCC 700603	Carbapenem resistant	Urine	7
MRSN 607201	Clinical isolate colistin sensitive	MRSN diversity panel	8
MRSN 430405	Clinical isolate colistin resistant	MRSN diversity panel	8

Supplemental References:

1. Bachmann BJ. Pedigrees of some mutant strains of Escherichia coli K-12. Bacteriol Rev. 1972;36(4):525-57. doi: 10.1128/br.36.4.525-557.1972. PubMed PMID: 4568763; PMCID: PMC408331.
2. Jacobs AC, Thompson MG, Black CC, Kessler JL, Clark LP, McQueary CN, Gancz HY, Corey BW, Moon JK, Si Y, Owen MT, Hallock JD, Kwak YI, Summers A, Li CZ, Rasko DA, Penwell WF, Honnold CL, Wise MC, Waterman PE, Lesho EP, Stewart RL, Actis LA, Palys TJ, Craft DW, Zurawski DV. AB5075, a Highly Virulent Isolate of Acinetobacter baumannii, as a Model Strain for the Evaluation of Pathogenesis and Antimicrobial Treatments. mBio. 2014;5(3):e01076-14. Epub 20140527. doi: 10.1128/mBio.01076-14. PubMed PMID: 24865555; PMCID: PMC4045072.
3. Gomez-Simmonds A, Uhlemann AC. Clinical Implications of Genomic Adaptation and Evolution of Carbapenem-Resistant Klebsiella pneumoniae. J Infect Dis. 2017;215(suppl_1):S18-s27. doi: 10.1093/infdis/jiw378. PubMed PMID: 28375514; PMCID: PMC5853309.
4. Podschun R, Ullmann U. Klebsiella spp. as nosocomial pathogens: epidemiology, taxonomy, typing methods, and pathogenicity factors. Clin Microbiol Rev. 1998;11(4):589-603. doi: 10.1128/cmr.11.4.589. PubMed PMID: 9767057; PMCID: PMC88898.
5. Wu KM, Li LH, Yan JJ, Tsao N, Liao TL, Tsai HC, Fung CP, Chen HJ, Liu YM, Wang JT, Fang CT, Chang SC, Shu HY, Liu TT, Chen YT, Shiau YR, Lauderdale TL, Su IJ, Kirby R, Tsai SF. Genome sequencing and comparative analysis of Klebsiella pneumoniae NTUH-K2044, a strain causing liver abscess and meningitis. J Bacteriol. 2009;191(14):4492-501. Epub 20090515. doi: 10.1128/jb.00315-09. PubMed PMID: 19447910; PMCID: PMC2704730.
6. Ramage B, Erolin R, Held K, Gasper J, Weiss E, Brittnacher M, Gallagher L, Manoil C. Comprehensive Arrayed Transposon Mutant Library of Klebsiella pneumoniae Outbreak Strain KPNIH1. J Bacteriol. 2017;199(20). Epub 20170919. doi: 10.1128/jb.00352-17. PubMed PMID: 28760848; PMCID: PMC5637181.
7. Rasheed JK, Anderson GJ, Yigit H, Queenan AM, Doménech-Sánchez A, Swenson JM, Biddle JW, Ferraro MJ, Jacoby GA, Tenover FC. Characterization of the extended-spectrum beta-lactamase reference strain, Klebsiella pneumoniae K6 (ATCC 700603), which produces the novel enzyme SHV-18. Antimicrob Agents Chemother. 2000;44(9):2382-8. doi: 10.1128/aac.44.9.2382-2388.2000. PubMed PMID: 10952583; PMCID: PMC90073.
8. Martin MJ, Stribling W, Ong AC, Maybank R, Kwak YI, Rosado-Mendez JA, Preston LN, Lane KF, Julius M, Jones AR, Hinkle M, Waterman PE, Lesho EP, Lebreton F, Bennett JW, Mc Gann PT. A panel of diverse Klebsiella pneumoniae clinical isolates for research and development. Microb Genom. 2023;9(5). doi: 10.1099/mgen.0.000967. PubMed PMID: 37141116; PMCID: PMC10272860.
